# Low-dose hydralazine during gestation reduces renal fibrosis in rodent offspring exposed to maternal high fat diet

**DOI:** 10.1371/journal.pone.0248854

**Published:** 2021-03-18

**Authors:** Benjamin P. Larkin, Sonia Saad, Sarah J. Glastras, Long T. Nguyen, Miao Hou, Hui Chen, Rosy Wang, Carol A. Pollock

**Affiliations:** 1 Renal Research Laboratory, Kolling Institute of Medical Research, University of Sydney, Sydney, Australia; 2 School of Life Sciences, Faculty of Science, University of Technology Sydney, Sydney, Australia; 3 Department of Diabetes, Endocrinology and Metabolism, Royal North Shore Hospital, Sydney, Australia; 4 Department of Cardiology, Children’s Hospital of Soochow University, Suzhou, Jiangsu, China; University Medical Center Utrecht, NETHERLANDS

## Abstract

**Background:**

Maternal high fat diet (HFD) promotes chronic kidney disease (CKD) in offspring. This is in accordance with the theory of fetal programming, which suggests adverse conditions occurring *in utero* predispose offspring to chronic conditions later in life. DNA methylation has been proposed as a key mechanism by which fetal programming occurs and is implicated in CKD progression. DNA demethylating drugs may interrupt the fetal programming of CKD by maternal obesity. Hydralazine, an antihypertensive agent, demethylates DNA at low doses which do not reduce blood pressure. We used a mouse model of maternal obesity to determine whether gestational administration of low-dose hydralazine to mothers can prevent CKD in offspring.

**Methods:**

C57BL/6 dams received HFD or chow from 6 weeks prior to mating and were administered subcutaneous hydralazine (5mg/kg) or saline thrice weekly during gestation. Male offspring were weaned to chow and were sacrificed at either postnatal week 9 or week 32. Biometric and metabolic parameters, renal global DNA methylation, renal structural and functional changes and markers of fibrosis, oxidative stress and inflammation were measured in offspring at weeks 9 and 32.

**Results:**

In week 9 offspring, maternal HFD consumption did not significantly alter anthropometric or metabolic parameters, or renal global DNA methylation. Week 32 offspring had increased renal global DNA methylation, together with albuminuria, glomerulosclerosis, renal fibrosis and oxidative stress. Administration of low-dose hydralazine to obese mothers during gestation reduced renal global DNA methylation and renal fibrotic markers in week 32 offspring.

**Conclusion:**

Gestational hydralazine reduced renal global DNA methylation in offspring of obese mothers and attenuated maternal obesity-induced renal fibrosis. These data support the use of low-dose hydralazine as a demethylating agent to prevent CKD arising in offspring due to maternal HFD consumption.

## Introduction

Fetal programming occurs when adverse exposures occurring *in utero* predispose the fetus to the development of disease later in life [1–3]. Maternal obesity is known to play a significant role in fetal programming and it is widely accepted that it increases offspring susceptibility to adult chronic conditions including cardiovascular disease, obesity, insulin resistance, type 2 diabetes (T2D), dyslipidaemia and hypertension [4–8]. We have previously shown that maternal obesity induced by a high fat diet (HFD) is also involved in the fetal programming of chronic kidney disease (CKD) [5, 9]. Using rodent models, we have observed that offspring of HFD-fed dams have significantly worse renal function, albuminuria and renal pathology including fibrosis, inflammation, and oxidative stress, compared with offspring of lean animals [5, 9].

Several mechanisms have been proposed in the fetal programming of CKD, and these include intrauterine environmental abnormalities in renal sympathetic activity, the renin-angiotensin system, sodium reabsorption, oxidative stress, inflammation and lipid pathways, which may be transmitted to the fetus [2, 3, 5, 7, 9, 10]. Epigenetic modifications, including DNA methylation, are heritable patterns regulating gene expression without altering the DNA sequence itself [11–13], and are also implicated in the developmental origins of CKD [2, 4, 14]. As most epigenetic signatures are laid down during the intrauterine period, coinciding with critical periods of increased fetal susceptibility to suboptimal maternal conditions [15–17], epigenetic modifications have been suggested as a key mechanism in fetal metabolic programming [18].

Associations between DNA methylation and CKD have been identified, providing insights into disease pathophysiology, severity and prognosis [19]. Importantly, DNA methylation has been shown to be significantly associated with the progression of renal fibrosis [20–23], the final common pathophysiological pathway in the development of end stage kidney disease [24]. As such, there is mounting interest in exploring the utility of DNA demethylating agents as potential novel therapies to prevent the progression of CKD. Under investigation for this purpose is the antihypertensive agent hydralazine, a drug which also possesses DNA demethylating effects [25, 26]. In murine models of renal fibrosis involving unilateral ureteral obstruction (UUO) and folic acid nephropathy, hydralazine ameliorated renal fibrosis by demethylating *RASAL1*, the gene encoding rasGAP-activating-like protein 1, which activates fibroblasts and induces renal fibrogenesis when its promoter is aberrantly methylated [20, 21, 23]. Interestingly, hydralazine was noted to exert its optimal demethylating activity at low doses, which are insufficient to cause lowering of blood pressure [23].

Given the role of DNA methylation in the fetal programming of CKD, it may be possible to treat mothers during pregnancy with hydralazine, to mitigate the adverse effects of maternal HFD consumption. Hydralazine has been used clinically for many decades in hypertensive disorders of pregnancy [27]. We hypothesised that low-dose hydralazine administered to obese mothers during pregnancy can reduce CKD development and progression in offspring. The aim of the present study was to use a mouse model of maternal HFD feeding to determine whether the demethylating agent hydralazine can interrupt the fetal programming of CKD by maternal HFD consumption, and elucidate the underlying mechanisms.

## Materials and methods

### a) Animal experiments

This study received ethics approval from the Animal Ethics Committee of Northern Sydney Local Health District (Approval Number Resp/17/29), and all work complied with the Australian code for the care and use of animals for scientific purposes [28]. Animal experiments were performed at the Kearns Animal Facility of the Kolling Institute of Medical Research, Royal North Shore Hospital, Sydney, Australia. All animals were weighed fortnightly, and general wellbeing observed thrice weekly. The model of maternal obesity was achieved by feeding 8-week old female C57BL/6 mice with a HFD (SF03-020, 20kJ/g, 23% fat, 43% of total energy from lipids, Specialty Feeds, WA, Australia); at 14 weeks of age, dams were co-housed with a chow-fed male for up to 72 hours, with pregnancy confirmed by the presence of a vaginal plug. Dams continued the same diet throughout gestation and lactation. The control group received a standard rodent chow diet (11kJ/g, 4.8% fat, 12% total energy from lipids, Specialty Feeds, WA, Australia) under the same experimental conditions. During gestation, dams from both the chow and HFD groups were allocated 1:1 to receive either subcutaneous hydralazine 5 mg/kg, a dose which does not lower blood pressure in mice [23], or normal saline 0.15 mL thrice weekly. Litter size was adjusted to four pups on postnatal day 1 to avoid differences in milk competition. Male offspring (on average 8 per group) were weaned to standard rodent chow on postnatal day 20; this yielded 4 offspring groups: control offspring (CC), lean offspring of obese mothers (HC), control offspring exposed to gestational hydralazine (CC+GH), and lean offspring of obese mothers exposed to gestational hydralazine (HC+GH). Offspring studies were performed in male offspring, rather than female, because CKD progression is more rapid in males [29], and male offspring are more susceptible to adverse fetal programming effects [30, 31].

One week prior to offspring sacrifice, blood pressure was measured using a non-invasive CODA® tail vein cuff method (Kent Scientific, CT, USA), and an intraperitoneal glucose tolerance test (IPGTT) was conducted. To perform IPGTT, animals were fasted for 5 hours after which a baseline blood glucose level was measured, then an intraperitoneal glucose injection (2g/kg, Phebra, Sydney, Australia) was delivered, and glucose measurements were taken from a tail nick at 15, 30, 60 and 90 minutes. The area under the curve (AUC) was calculated. All blood glucose measurements were performed using a glucometer (Accu-Chek®, Roche Diagnostics, NSW, Australia).

Offspring were sacrificed under fasting conditions either during adolescence (postnatal week 9), or later in adulthood at 32 weeks of age. Deep anaesthesia was achieved using isoflurane, then blood was collected via cardiac puncture, and a urine sample obtained by bladder puncture. Phosphate-buffered saline was used to perfuse the body, and the kidneys, liver and fat were harvested. Organs were either fixed in 10% formalin solution for histological analysis, or snapped frozen for protein quantification, RNA or DNA extraction.

### b) Serum measures

Serum insulin was measured (n = 5) using the Ultra Sensitive Mouse Insulin ELISA Kit (Crystal Chem, IL, USA), and the insulin resistance index (HOMA-IR) was calculated using the formula: fasting insulin (mU/L) x fasting glucose (mmol/L)/22.5. A colorimetric assay kit was used to measure serum creatinine (Cayman Chemical, MI, USA) (n = 8).

### c) Urine measures

A mouse albumin ELISA kit (Crystal Chem) was used to quantify urinary albumin concentration, and creatinine concentration was determined using a colorimetric assay kit (Cayman Chemical). The urinary albumin:creatinine ratio was then calculated (n = 5–8).

### d) Global DNA methylation analysis

The ISOLATE II Genomic DNA Kit (Bioline, London, UK) was used to extract genomic DNA from kidney cortex samples (n = 4–5). Following this, 5-methylcytosine (5mC) percentage of total DNA was calculated using the Methylated DNA Quantification Kit (Abcam).

### e) Renal structural changes

Formalin-fixed kidneys were sectioned (2 μm thickness) and Periodic Acid Schiff (PAS) staining was performed (6 samples per group). Light microscopy (Leica, Germany) was used to assess for glomerulosclerosis, scored by two independent, blinded investigators. Glomeruli were graded on a scale of 0 to 4 as follows: (0: normal; 1: involvement of <25% of the glomerulus; 2: involvement of 25–50% of the glomerulus; 3: involvement of 50–75% of the glomerulus; 4: totally sclerosed) [5, 32]. The glomerulosclerosis index was calculated by averaging the scores of 20 glomeruli per slide. Formalin-fixed kidney sections (n = 6) were also stained by picrosirius red, to identify collagens I and III, known markers of renal fibrosis. Four non-overlapping images of kidney cortex were captured using a digital camera attached to the light microscope (Leica Application Suite, Leica, Germany), and quantified using Image J software (National Institutes of Health, USA). Kidney specimens were also assessed by two independent blinded investigators for tubulointerstitial fibrosis, tubular vacuolation, tubular dilatation and casts, as previously described [32].

### f) Immunohistochemistry

Formalin-fixed kidneys (6 samples per group) were sectioned (4 μm thickness) and placed on slides, which were deparaffinised, hydrated and underwent heat retrieval in a water bath at 99°C for 20 mins using 0.01M citrate buffer, pH 6. Slides were blocked with Protein Block Serum-Free (Dako, Glostrup, Denmark), and were incubated at 4°C overnight with the following primary antibodies: fibronectin (dilution 1:1000, Abcam, Cambridge, UK), collagen IV (1:750, Abcam), Collagen III (1:750, Abcam), 8-hydroxy-2’-deoxyguanosine (8-OHdG) (1:750, Bioss, MA, USA), and nitrotyrosine (1:500, Merck Millipore Ltd, Darmstadt, Germany). Slides then underwent incubation with horseradish peroxidase anti-rabbit Envision system (Dako, Japan), and were stained with 3,3’-diaminobenzidine tetrahydrochloride and counterstained with Mayer’s haematoxylin. A digital camera attached to a microscope captured 4–6 non-overlapping images at 200x magnification, prior to Image J software being utilised to objectively quantitate the area stained.

### g) Protein extraction and western blotting

Funato lysis buffer (50mM Tris-HCl pH 7.4, 150 mM NaCl, 1mM EDTA pH 8, 1% Triton X-100, Roche protease inhibitor) was used to aid homogenisation of kidney cortex by a TissueRuptor (Qiagen, Hilden, Germany). The resulting homogenate was centrifuged at 10,000g for 15 min and the supernatant protein concentration was determined with the Pierce BCA Protein Kit (Thermo Fisher Scientific, VIC, Australia). 20 μg of protein was loaded onto an Invitrogen Bolt 4–12% Bis-Tris Plus Gel (Thermo Fisher Scientific) for electrophoresis (n = 6), before transfer to a Hybond Nitrocellulose membrane (Amersham Pharmacia Biotech, Bucks, UK). Incubation of membranes at 4°C overnight was performed using the following primary antibodies: fibronectin (dilution 1:2000, Thermo Fisher Scientific), collagen III (1:2500, Abcam, Cambridge, UK), collagen IV (1:5000, Abcam), manganese superoxide dismutase (MnSOD 1:2000, Merck Millipore Ltd, Darmstadt, Germany), and α-tubulin (1:2000, Sigma-Aldrich, MO, USA). Membranes then underwent incubation with a horseradish peroxidase-conjugated secondary antibody, and were developed using ImageQuant LAS 4000 (Fujifilm, Tokyo, Japan). GelPro Analyser software (Informer Technologies Inc.) was used for analysis. Protein expression was expressed as a proportion relative to α-tubulin, a commonly used internal control.

### h) Quantitative real-time PCR (RT-PCR)

The RNeasy Plus Mini Kit (Qiagen, CA, USA) was used to extract RNA from kidney cortical tissue (6 samples per group). Synthesis of cDNA was subsequently performed using the iScript cDNA Synthesis Kit (Biorad, CA, USA). RT-PCR was carried out with the QuantStudio 12K Flex Real-Time PCR System (Thermo Fisher Scientific), using QuantiNova SYBR Green (Qiagen) mastermixes and PCR primers listed in Table 1. Results were normalised to 18S and expressed as fold change.

**Table 1:** Mouse specific primers used in quantitative real-time PCR.

Gene	Forward Primer Sequence	Reverse Primer Sequence
18S	ACCGCAGCTAGGAATAATGGA	GCCTCAGTTCCGAAAACC
catalase	CTCCATCAGGTTTGTTTCTTG	CAACAGGCAAGTTTTTGATG
CD68	CAATTCAGGGTGGAAGAAAG	TCTGATGTAGGTCCTGTTTG
DNMT1	GTGAACAGGAGATGACAAC	CTGGATCCTCCTTTGATTTC
DNMT3a	ACCAGAAGAAGAGAAGAATCC	CAATGATCTCCTTGACCTTAG
collagen I	CATGTTCAGCTTTGTGGACCT	GCAGCTGACTTCAGGGATGT
collagen III	TCCCCTGGAATCTGTGAATC	TGAGTCGAATTGGGGAGAAT
collagen IV	AAGGACTCCAGGGACCAC	CCCACTGAGCCTGTCACAC
Fibronectin	CGGAGAGAGTGCCCCTACTA	CGATATTGGTGAATCGCAGA
MCP-1	GCCTGCTGTTCACAGTTGC	CAGGTGAGTGGGGCGTTA
Nox2	CTACCTAAGATAGCAGTTGATG	TACCAGACAGACTTGAGAATG
Tet3	AGGATCGGTATGGAGAAAAG	CAGGATCAAGATAACAATCACG

### i) Statistical analysis

Results are expressed as mean ± SEM. Statistical analysis occurred either using analysis of variance (ANOVA) followed by Tukey’s post hoc test, or using unpaired t-tests. GraphPad Prism software v8.3.1 (San Diego, CA, USA) was used. Significant results had p values of <0.05.

## Results

### Biometric parameters

**Fig 1:**
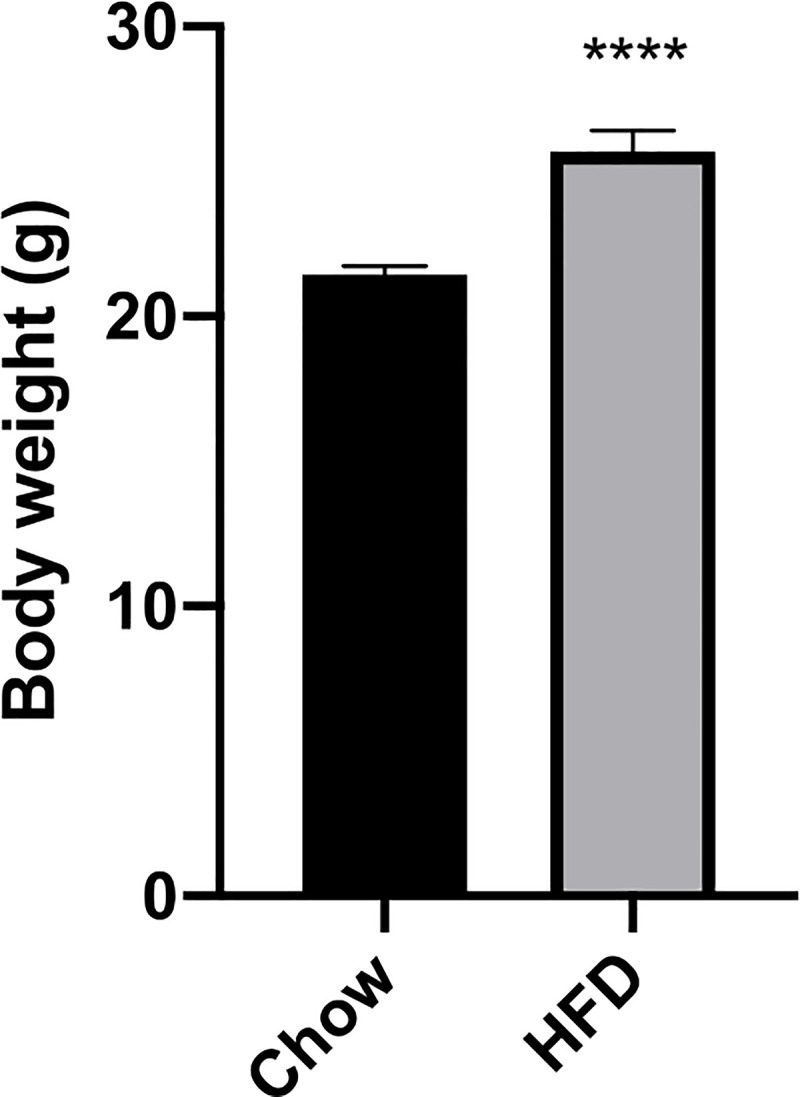
Maternal body weight at the commencement of breeding. Pre-breeding body weight of mothers. Results expressed as mean ± SEM, n = 13–20. ****p<0.0001.

At the commencement of breeding, HFD-fed dams had significantly higher body weights compared to dams fed chow diet (p<0.0001, Fig 1). There were no significant differences in body weight of obese dams that received hydralazine versus saline injections prior to conception.

At postnatal week 9, there were no significant differences in body weight between offspring born to obese versus lean mothers, and hydralazine treatment of dams during gestation did not affect offspring body weight (Table 2). Offspring kidney weight relative to total body weight was unaffected by the presence of maternal obesity or hydralazine treatment during gestation. As a proportion of total body weight, liver weight was significantly higher in HC compared to CC (p<0.05, Table 2), which remained unaffected by the presence of hydralazine. Relative to total body weight, epididymal and retroperitoneal fat weights were similar between groups (Table 2). Mean blood pressure at postnatal week 9 was similar between offspring groups.

**Table 2:** Biometric parameters of week 9 offspring.

	CC	HC	CC+GH	HC+GH
Body weight (g)	25.46 ± 0.56	24.18 ± 0.33	24.56 ± 0.54	23.55 ± 0.32
Kidney weight (g)	0.19 ± 0.01	0.19 ± 0.01	0.18 ± 0.01	0.18 ± 0.00
Kidney weight/body weight (%)	0.75 ± 0.04	0.77 ± 0.04	0.72 ± 0.02	0.77 ± 0.02
Liver weight (g)	1.26 ± 0.04	1.42 ± 0.07	1.18 ± 0.03	1.35 ± 0.07 #
Liver weight/ body weight (%)	4.98 ± 0.18	5.86 ± 0.32 *	4.83 ± 0.17	5.76 ± 0.38 #
Epididymal fat weight (g)	0.35 ± 0.02	0.33 ± 0.03	0.29 ± 0.02	0.36 ± 0.02
Epididymal fat weight/body weight (%)	1.40 ± 0.09	1.35 ± 0.12	1.18 ± 0.09	1.52 ± 0.09 #
Retroperitoneal fat weight (g)	0.07 ± 0.00	0.06 ± 0.01	0.06 ± 0.00	0.05 ± 0.01
Retroperitoneal fat weight/ body weight (%)	0.07 ± 0.00	0.06 ± 0.01	0.06 ± 0.00	0.05 ± 0.01
Mean BP (mmHg)	83.97 ± 2.63	82.60 ± 7.65	88.88 ± 4.17	90.28 ± 2.86

Results expressed as mean ± SEM, n = 5–10

CC vs HC

*p<0.05

CC+GH vs HC+GH

# p<0.05

In mid adulthood, postnatal week 32, offspring weight was unaffected by exposure to maternal obesity or gestational hydralazine (Table 3). The kidney/total body weight ratio was significantly higher in HC than in CC (p<0.01, Table 3), and the relationship between maternal diet and offspring kidney/total body weight ratio was preserved in the presence of gestational hydralazine (HC+GH vs. CC+GH, p<0.05, Table 3). The liver/total body weight ratio was significantly higher in offspring of obese mothers than in controls (p<0.05), however, liver/total body weight was similar between HC and HC+GH (Table 3). Epididymal fat weight relative to total body weight was significantly lower in HC compared to CC (p<0.05), however, this effect was not observed in the presence of gestational hydralazine (Table 3). Retroperitoneal fat/total body weight ratio was similar in offspring of obese mothers and controls and was unaffected by gestational hydralazine (Table 3). At postnatal week 32, there were no significant differences in mean blood pressure between offspring groups (Table 3).

**Table 3:** Biometric parameters of week 32 offspring.

	CC	HC	CC+GH	HC+GH
Body weight (g)	34.65 ± 1.04	33.47 ± 0.67	35.16 ± 0.94	33.70 ± 0.67
Kidney weight (g)	0.25 ± 0.00	0.29 ± 0.01 *	0.28 ± 0.01	0.33 ± 0.03
Kidney weight/body weight (%)	0.72 ± 0.02	0.87 ± 0.03 **	0.78 ± 0.03	0.99 ± 0.10 #
Liver weight (g)	1.39 ± 0.06	1.48 ± 0.06	1.52 ± 0.03	1.59 ± 0.04
Liver weight/ body weight (%)	3.89 ± 0.12	4.41 ± 0.15 *	4.28 ± 0.15	4.73 ± 0.14
Epididymal fat weight (g)	0.89 ± 0.11	0.55 ± 0.09 *	0.79 ± 0.09	0.64 ± 0.05
Epididymal fat weight/body weight (%)	2.53 ± 0.24	1.62 ± 0.25 *	2.18 ± 0.22	1.91 ± 0.15
Retroperitoneal fat weight (g)	0.34 ± 0.06	0.20 ± 0.07	0.33 ± 0.04	0.20 ± 0.03 #
Retroperitoneal fat weight/ body weight (%)	0.81 ± 0.18	0.59 ± 0.19	0.92 ± 0.09	0.62 ± 0.10
Mean BP (mmHg)	83.24 ± 2.52	89.98 ± 4.48	86.11 ± 3.30	95.68 ± 4.11

Results expressed as mean ± SEM, n = 6–8

CC vs HC

*p<0.05

**p<0.01

CC+GH vs HC+GH

# p<0.05

### Metabolic markers

In offspring at postnatal week 9, similar levels of glucose tolerance were observed irrespective of maternal obesity or hydralazine administration during gestation (Table 4). Similarly, neither maternal obesity nor hydralazine impacted glucose tolerance in offspring at 32 weeks (Table 5). Pre-terminal fasting blood glucose levels were similar between offspring groups at week 9, and week 32 (Tables 4 and 5). At both weeks 9 and 32, there were no group differences in fasting serum insulin concentrations or HOMA-IR, a measure of insulin resistance (Tables 4 and 5). Renal function, as measured by serum creatinine, was similar between groups at weeks 9 and 32 (Tables 4 and 5).

**Table 4:** Metabolic parameters of week 9 offspring.

	CC	HC	CC+GH	HC+GH
IPGTT AUC	1595 ± 56	1600 ± 26	1693 ± 48	1809 ± 38
Glucose (mmol/L)	15.98 ± 0.40	17.60 ± 0.96	13.96 ± 0.90	14.73 ± 1.39
Serum insulin (ng/mL)	0.32 ± 0.12	0.42 ± 0.15	0.41 ± 0.20	0.29 ± 0.07
HOMA-IR	2.91 ± 1.15	4.16 ± 1.61	3.65 ± 1.76	2.84 ± 0.59
Serum creatinine (μmol/L)	76.62 ± 14.38	74.52 ± 18.11	85.35 ± 12.69	50.71 ± 10.12
Urinary albumin:creatinine ratio (μg/mg)	9.22 ± 1.56	16.86 ± 4.52	14.84 ± 3.22	16.03 ± 1.83

Results expressed as mean ± SEM

**Table 5:** Metabolic parameters of week 32 offspring.

	CC	HC	CC+GH	HC+GH
IPGTT AUC	1657 ± 66	1586 ± 77.07	1615 ± 111	1585 ± 59
Glucose (mmol/L)	11.89 ± 0.80	12.65 ± 1.09	13.07 ± 0.67	11.61 ± 0.72
Serum insulin (ng/mL)	0.50 ± 0.14	0.25 ± 0.05	0.40 ± 0.13	0.30 ± 0.06
HOMA-IR	5.97 ± 1.42	3.28 ± 0.97	5.07 ± 1.85	3.43 ± 0.90
Serum creatinine (μmol/L)	81.20 ± 16.12	58.15 ± 16.93	66.83 ±12.68	69.37 ± 17.19
Urinary albumin:creatinine ratio (μg/mg)	8.15 ± 2.30	15.88 ± 2.46 *	10.48 ± 2.59	14.65 ± 3.14

Results expressed as mean ± SEM

CC vs HC

*p<0.05

### Maternal obesity induced albuminuria in week 32 offspring; gestational hydralazine was not protective

At week 9, urinary albumin:creatinine ratio was unaffected by maternal obesity or gestational hydralazine (Table 4). By week 32, offspring of obese mothers had significantly increased albuminuria compared to controls (p<0.05, Table 5). Hydralazine administration during gestation did not significantly attenuate albuminuria in the offspring of obese mothers (Table 5).

### Effect of maternal obesity and gestational hydralazine on DNA methylation in offspring

**Fig 2:**
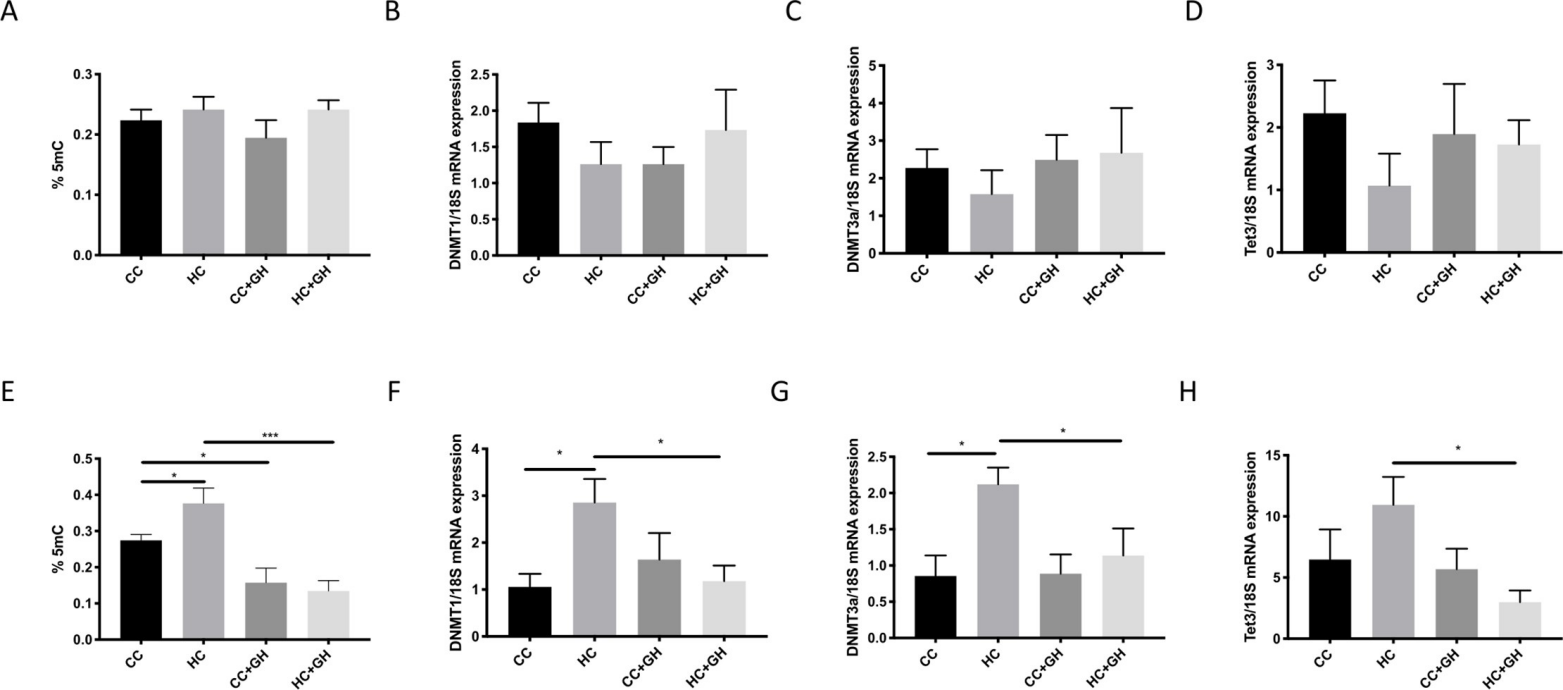
DNA methylation markers. (A) Renal global DNA methylation expressed as % 5mC of total DNA in week 9 offspring, (B-D) week 9 offspring mRNA expression of DNMT1, DNMT3a and Tet3, (E) renal global DNA methylation expressed as % 5mC of total DNA in week 32 offspring, (F-H) week 32 offspring mRNA expression of DNMT1, DNMT3a and Tet3. Results expressed as mean ± SEM, n = 4–6. *p<0.05, ***p<0.001.

At week 9, there were no significant differences between offspring groups in renal global DNA methylation (Fig 2A). Further, renal mRNA expression of DNA methyltransferases (DNMT)-1 and DNMT3a were similar between offspring of obese, compared to lean, mothers. Hydralazine administration to mothers during gestation did not significantly alter mRNA expression of DNMTs in offspring kidneys at week 9 (Fig 2B and 2C). There were no significant differences between offspring groups in the mRNA expression of the enzyme ten-eleven-translocation (Tet)-3 at week 9 (Fig 2D).

At week 32, offspring exposed to maternal obesity had increased renal global DNA methylation compared to controls (HC vs CC, p<0.05, Fig 2E). Gestational hydralazine was associated with decreased global methylation in offspring of chow-fed mothers (CC+GH vs. CC, p<0.05), and in offspring of HFD-fed mothers (HC+GH vs. HC, p<0.001, Fig 2E). Renal mRNA expression of DNMT1 was significantly elevated in offspring of obese mothers (HC vs. CC, p<0.05, Fig 2F). Gestational hydralazine significantly reduced DNMT1 mRNA expression in offspring of obese mothers (HC+GH vs. HC p<0.05). Similarly, offspring of obese mothers had significantly elevated renal DNMT3a expression compared with control, at week 32 (HC vs. CC, p<0.05, Fig 2G). Gestational administration of hydralazine significantly attenuated DNMT3a mRNA expression in offspring of HFD-fed mothers (HC+GH vs. HC, p<0.05).

While renal Tet3 mRNA expression was similar between CC and HC, hydralazine administration significantly reduced renal Tet3 mRNA expression in offspring of obese mothers (HC+GH vs. HC p<0.05, Fig 2H).

### Renal structural changes

**Fig 3:**
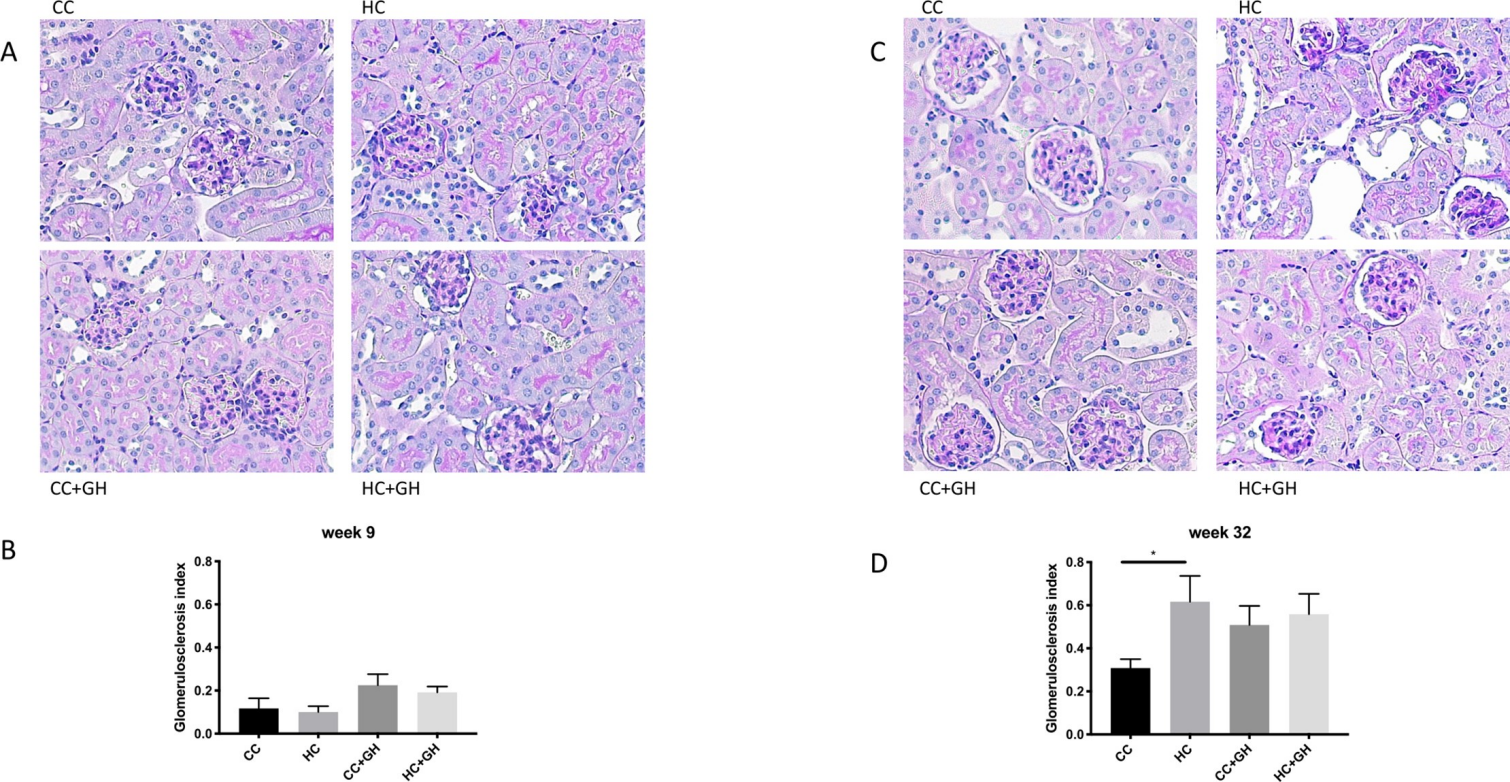
Glomerulosclerosis. (A, B) Week 9 offspring representative images of PAS staining for glomerulosclerosis at 400x magnification, and glomerulosclerosis index, (C, D) week 32 offspring representative images of PAS staining for glomerulosclerosis at 400x magnification, and glomerulosclerosis index. Results expressed as mean ± SEM, n = 6. *p<0.05.

PAS staining of kidney tissue from offspring at week 9 demonstrated only a mild degree of glomerulosclerosis across all groups, with no significant differences between groups (Fig 3A and 3B). Similarly, at postnatal week 32, offspring had evidence of mild glomerulosclerosis overall, though HC had an increased glomerulosclerosis index when compared to CC (p<0.05, Fig 3C and 3D). Hydralazine administration did not reduce glomerulosclerosis in offspring of either HFD or chow-fed mothers.

**Fig 4:**
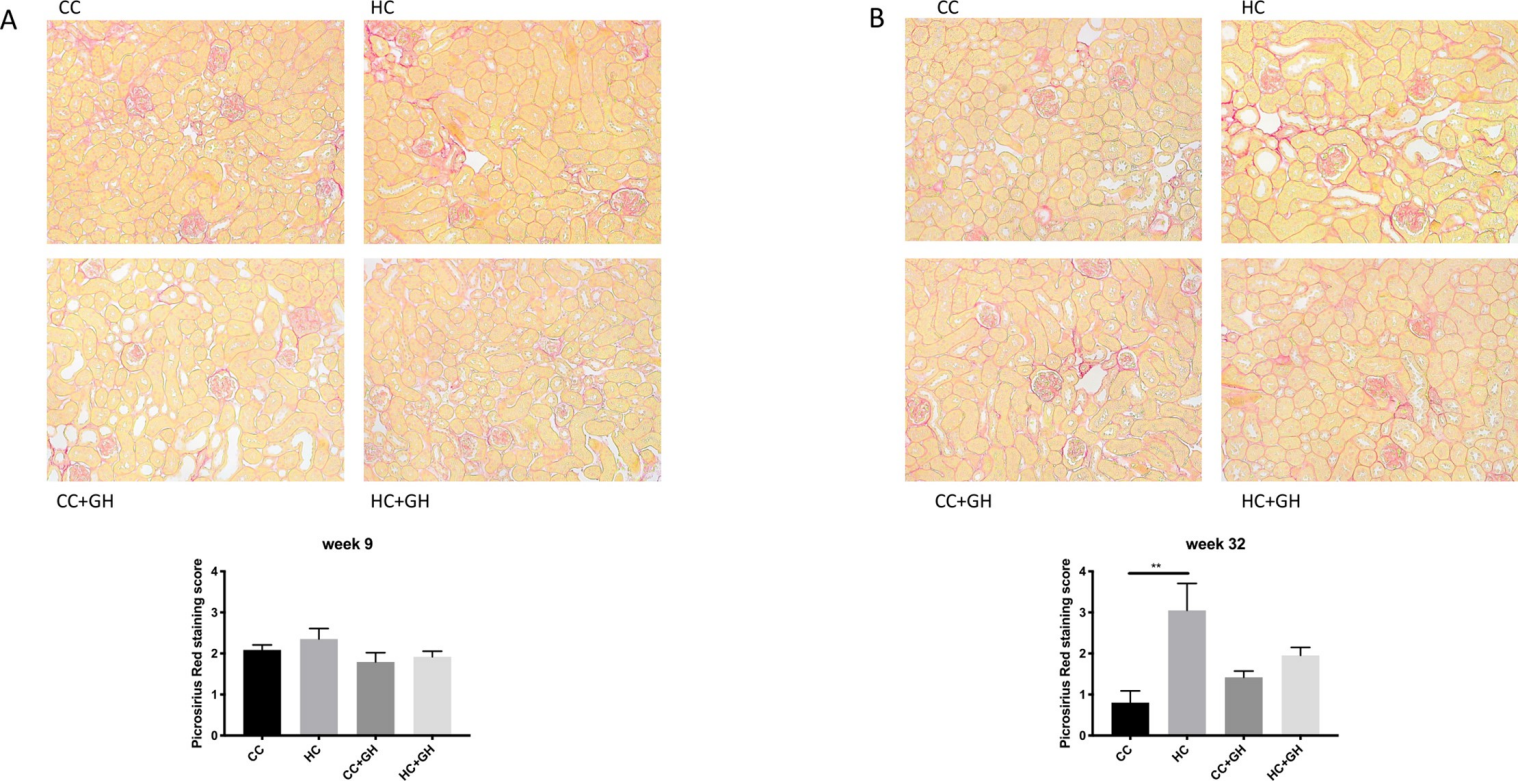
Renal fibrosis as measured by Picrosirius Red (PSR) staining. (A) Week 9 offspring representative images of PSR staining at 200x magnification, and quantitation, (B) week 32 offspring representative images of PSR staining at 200x magnification, and quantitation. Results expressed as mean ± SEM, n = 5–6, **p<0.01.

Picrosirius red staining of kidney tissue from week 9 offspring did not demonstrate any significant differences between groups (Fig 4A). At week 32, there was increased staining in HC compared to CC (Fig 4B, p<0.01), however gestational hydralazine did not attenuate picrosirius red staining in offspring of obese mothers.

**Fig 5:**
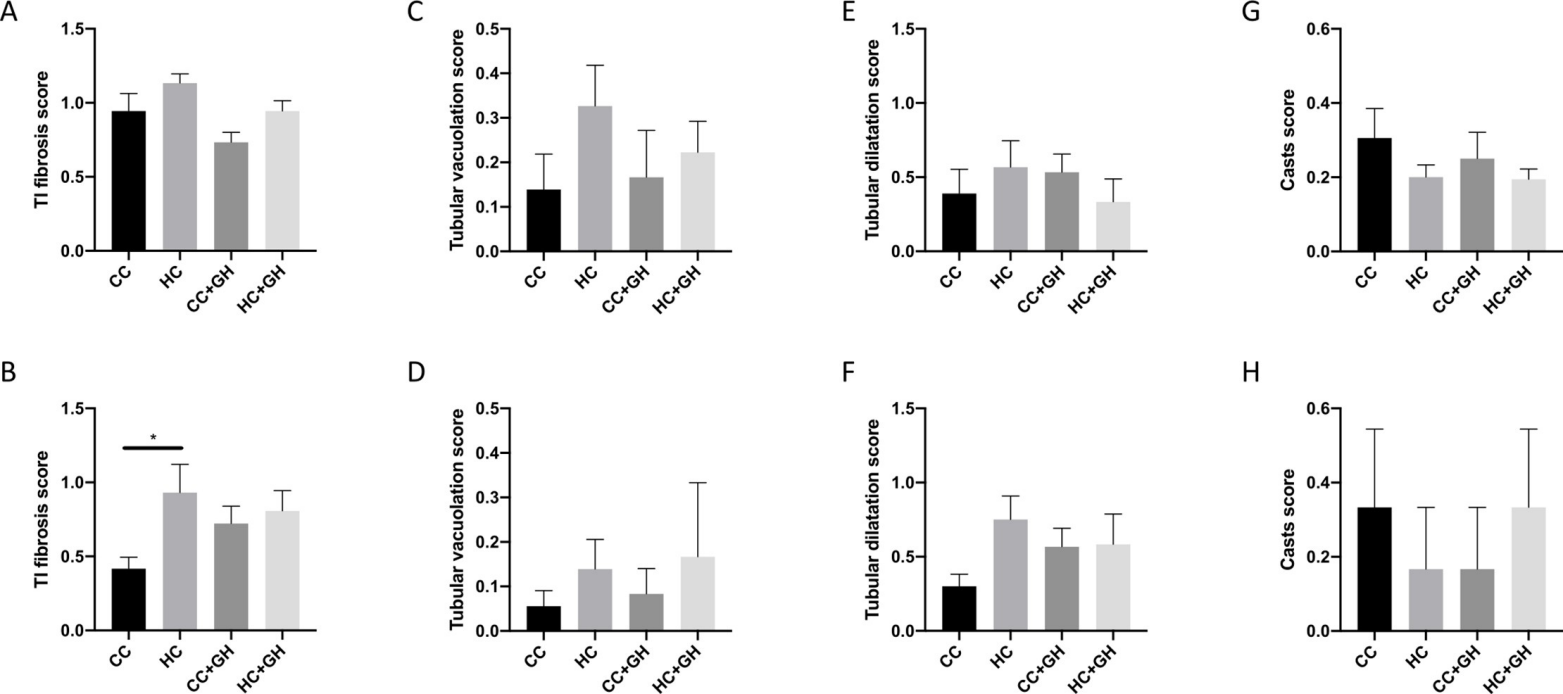
Renal structural changes. Kidneys assessed by PAS and Picrosirius Red staining. (A, B) Tubulointerstitial fibrosis assessment at week 9 and week 32, (C, D) tubular vacuolation assessment at week 9 and week 32, (E, F) tubular dilatation assessment at week 9 and week 32, (G, H) casts scoring at week 9 and week 32. Results expressed as mean ± SEM, n = 6. *p<0.05.

Tubulointerstitial fibrosis was similar between HC and CC at week 9 (Fig 5A). At week 32, there was increased tubulointerstitial fibrosis in HC compared to CC (p<0.05, Fig 5B). Tubular vacuolation was similar between groups at both weeks 9 and 32 (Fig 5C and 5D). No significant differences in tubular dilatation were observed between groups at week 9 and at week 32 (Fig 5E and 5F). At both timepoints, casts were similar between groups (Fig 5G and 5H). Gestational hydralazine did not significantly affect tubulointerstitial fibrosis, tubular vacuolation, tubular dilatation or casts in offspring at either week 9 or week 32 (Fig 5A–5H).

### Gestational hydralazine reduced renal fibrosis markers in offspring of obese mothers

**Fig 6:**
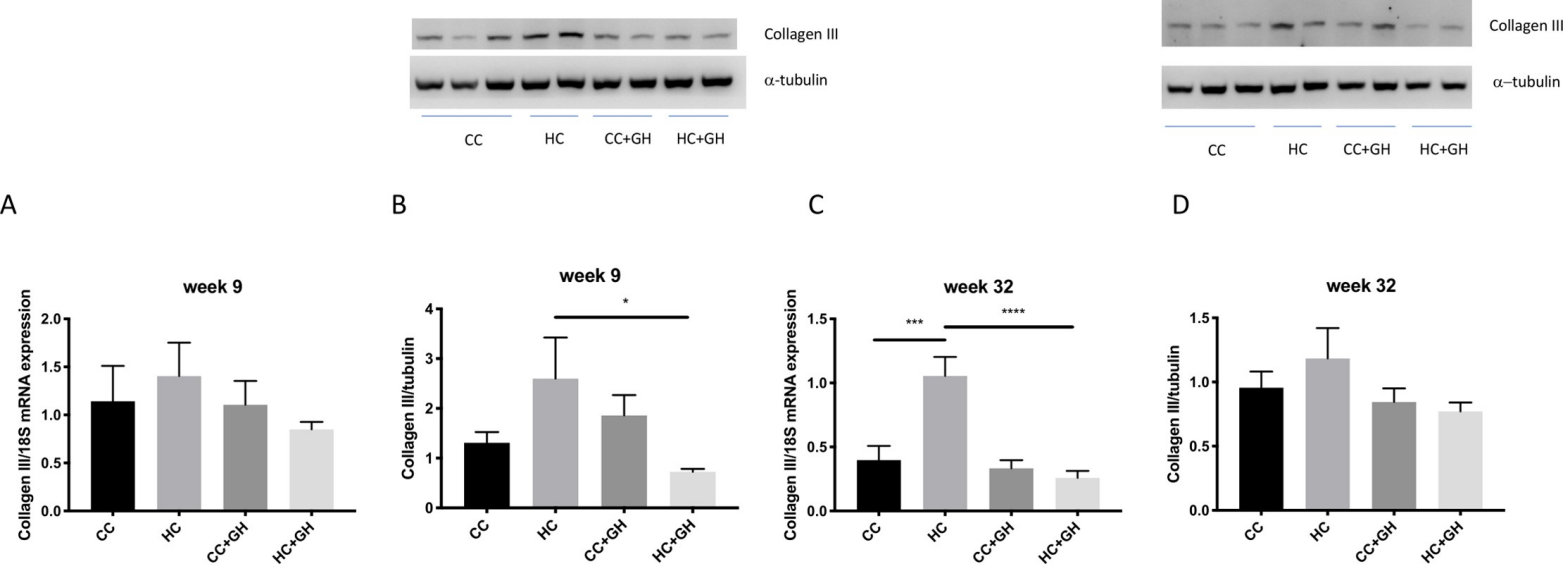
Renal fibrotic changes as measured by collagen III. (A) mRNA expression of collagen III at week 9, (B) western blotting for collagen III at week 9, (C) mRNA expression of collagen III at week 32, (D) western blotting for collagen III at week 32. Results expressed as mean ± SEM, n = 6. *p<0.05, ***p<0.001, ****p<0.0001.

**Fig 7:**
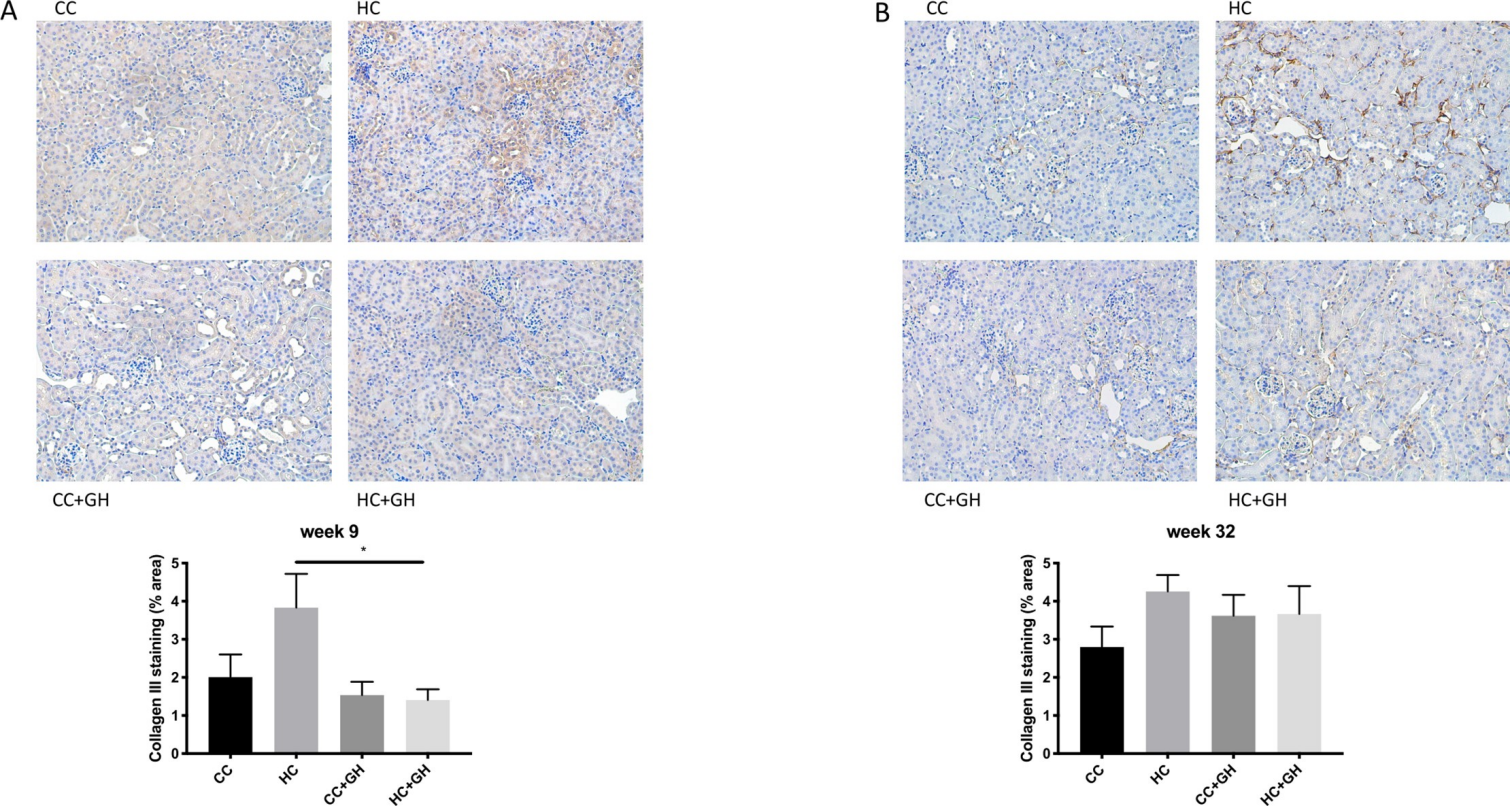
Renal fibrotic changes as measured by collagen III immunohistochemistry. (A) Week 9 offspring representative images of collagen III staining at 200x magnification, and quantitation (B) Week 32 offspring representative images of collagen III staining at 200x magnification, and quantitation. Results expressed as mean ± SEM, n = 6. *p<0.05.

**Fig 8:**
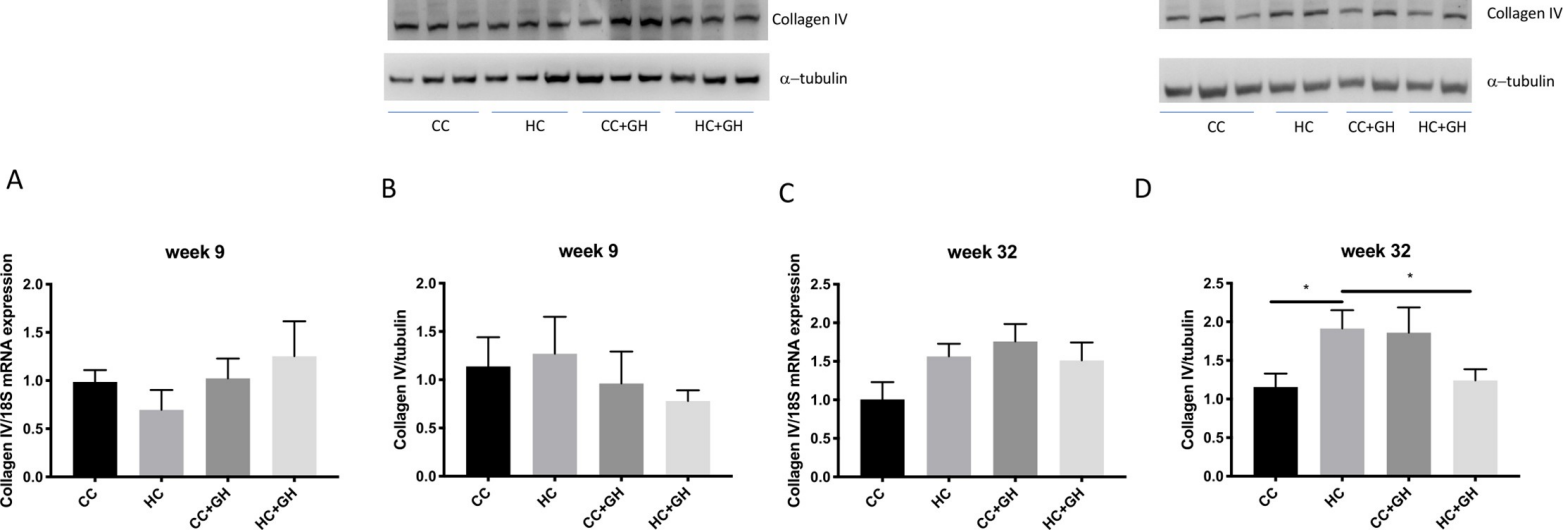
Renal fibrotic changes as measured by collagen IV. (A) Week 9 offspring mRNA expression of collagen IV, (B) week 9 offspring western blotting for collagen IV, (C) week 32 offspring mRNA expression of collagen IV, (D) week 32 offspring western blotting for collagen IV. Results expressed as mean ± SEM, n = 6. *p<0.05.

**Fig 9:**
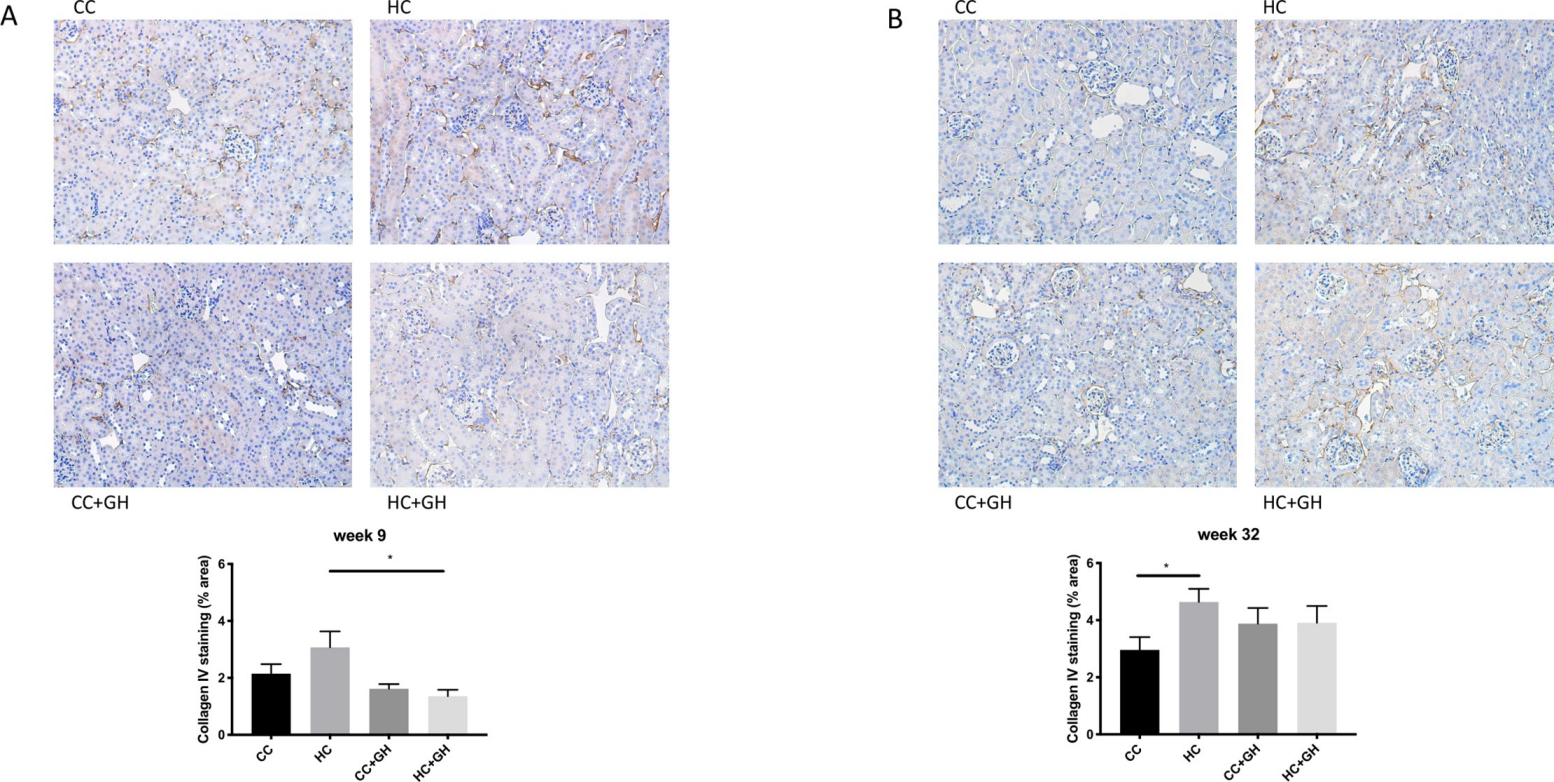
Renal fibrotic changes as measured by collagen IV immunohistochemistry. (A) Week 9 offspring representative images of collagen IV staining at 200x magnification, and quantitation (B) week 32 offspring representative images of collagen IV staining at 200x magnification, and quantitation. Results expressed as mean ± SEM, n = 6. *p<0.05.

**Fig 10:**
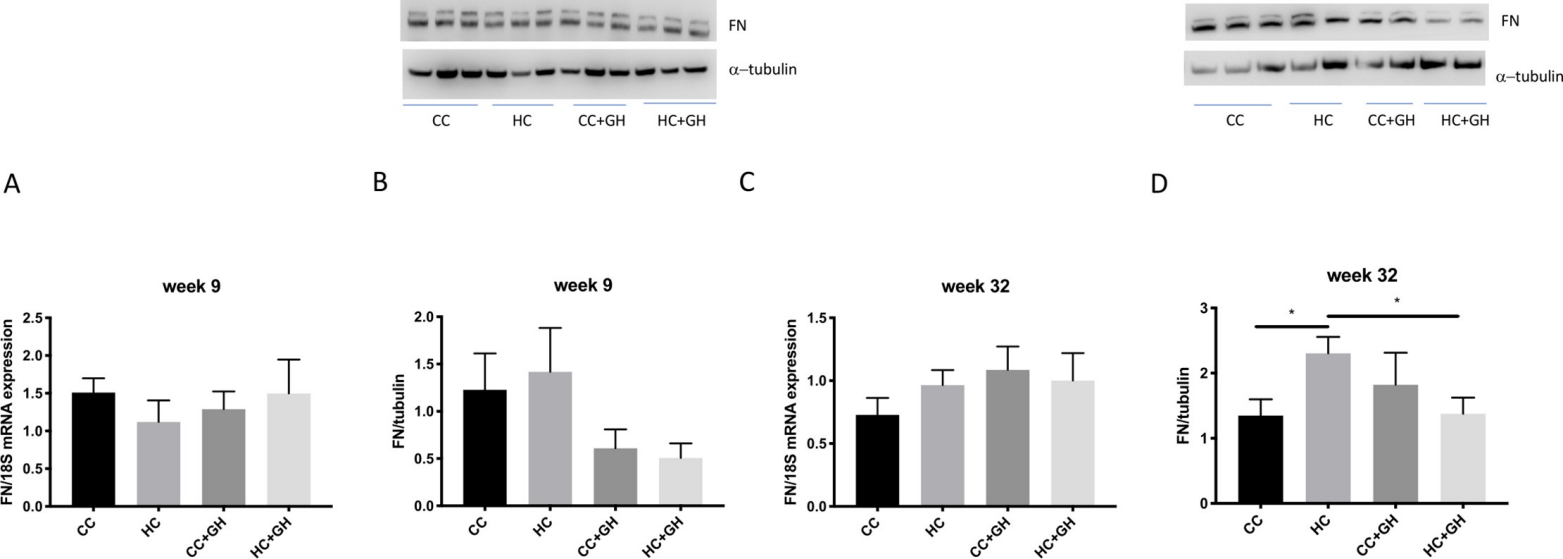
Renal fibrotic changes as measured by fibronectin (FN). (A) Week 9 offspring mRNA expression of FN, (B) week 9 offspring western blotting for FN, (C) week 32 offspring mRNA expression of FN, (D) week 32 offspring western blotting for FN. Results expressed as mean ± SEM, n = 6. *p<0.05.

**Fig 11:**
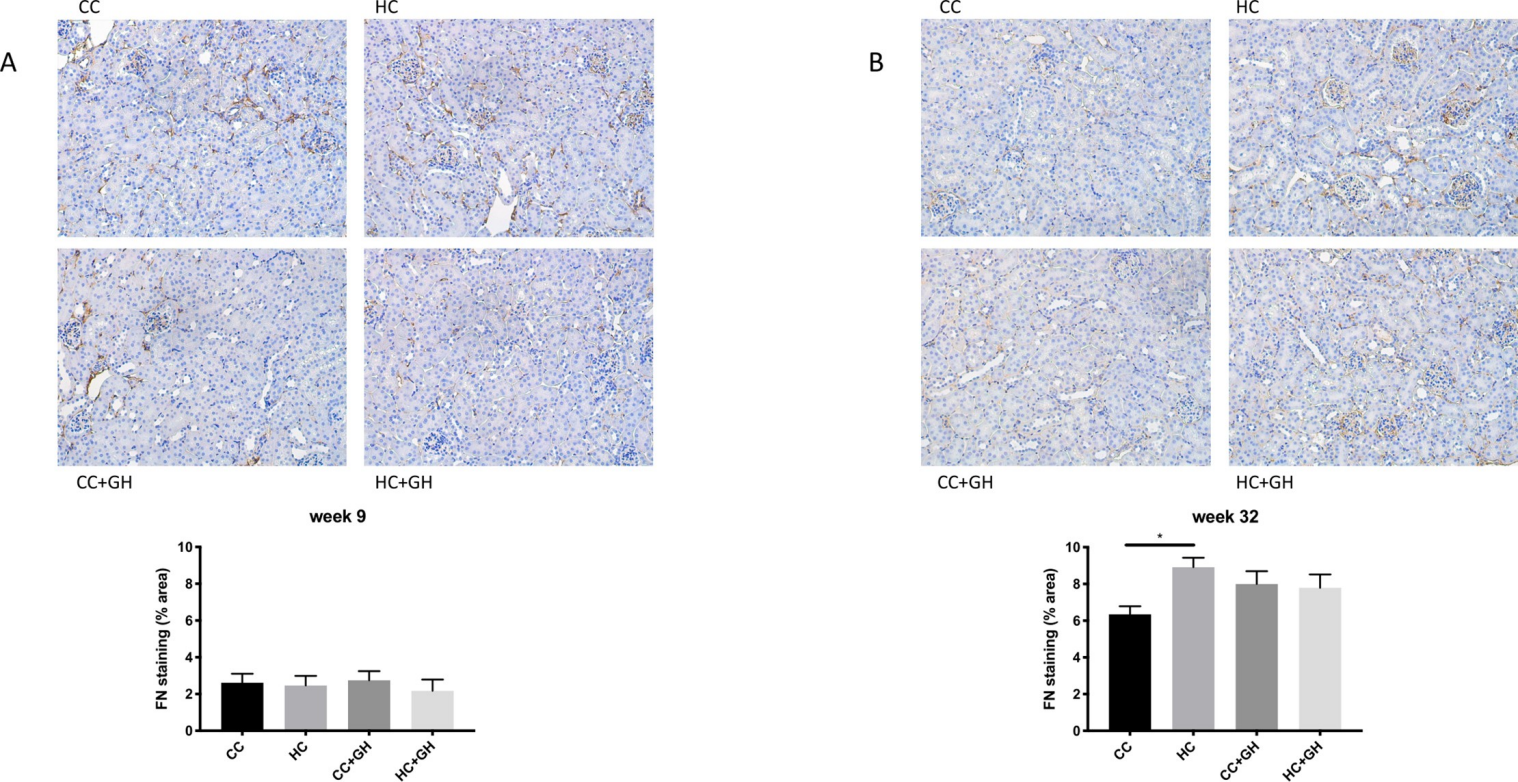
Renal fibrotic changes as measured by fibronectin (FN) immunohistochemistry. (A) Week 9 offspring representative images of FN staining at 200x magnification, and quantitation (B) week 32 offspring representative images of FN staining at 200x magnification, and quantitation. Results expressed as mean ± SEM, n = 6, *p<0.05.

Extracellular matrix components collagen III, collagen IV and fibronectin were used to assess for renal fibrosis. At week 9, renal mRNA expression of collagen III was similar in all offspring groups (Fig 6A). Protein expression of collagen III was similar between HC and CC, however there was a protective effect of gestational hydralazine, with significantly reduced collagen III expression in offspring of obese mothers administered hydralazine in pregnancy (western blotting: HC+GH vs. HC, p<0.05, Fig 6B; immunohistochemistry: HC+GH vs. HC, p<0.05, Fig 7A). At week 9, renal collagen IV mRNA and protein expression were similar between HC and CC (Figs 8A, 8B and 9A). Hydralazine treatment resulted in a significant reduction in collagen IV protein expression in offspring of obese mothers (HC+GH vs. HC, p<0.05, Fig 9A). There were no differences between groups in fibronectin mRNA expression at week 9 (Fig 10A). Fibronectin protein expression was similar between groups by western blotting (Fig 10B) and immunohistochemistry (Fig 11A).

At postnatal week 32, the effect of maternal obesity on renal fibrosis was more pronounced in offspring. For collagen III, there was a marked increase in mRNA expression in the kidneys of HC compared to CC (p<0.001, Fig 6C), and gestational hydralazine attenuated collagen III mRNA expression due to maternal obesity (HC+GH vs HC, p<0.0001). Collagen III protein expression tended to increase in HC compared to CC, however, gestational hydralazine did not significantly reduce collagen III protein expression in HC+GH compared to HC (Figs 6D and 7B). There was a trend towards increased collagen IV mRNA expression in HC compared to CC (p = 0.07), although hydralazine exposure did not reduce collagen IV mRNA expression in HC+GH compared to HC (Fig 8C). There was a significant increase in collagen IV protein expression in HC compared to CC (p<0.05, Figs 8D and 9B). With exposure to gestational hydralazine, no differences in collagen IV protein expression were detected between offspring of chow-fed and HFD-fed mothers (CC+GH vs. HC+GH, Figs 8D and 9B). Fibronectin mRNA expression in kidneys was similar between all offspring groups (Fig 10C). Renal expression of fibronectin protein was higher in HC than CC (p<0.05, Figs 10D and 11B). Gestational hydralazine ameliorated this effect, with no significant differences observed in fibronectin protein expression between CC+GH and HC+GH (Figs 10D and 11B).

### Effect of maternal obesity and gestational hydralazine on offspring renal oxidative stress

**Fig 12:**
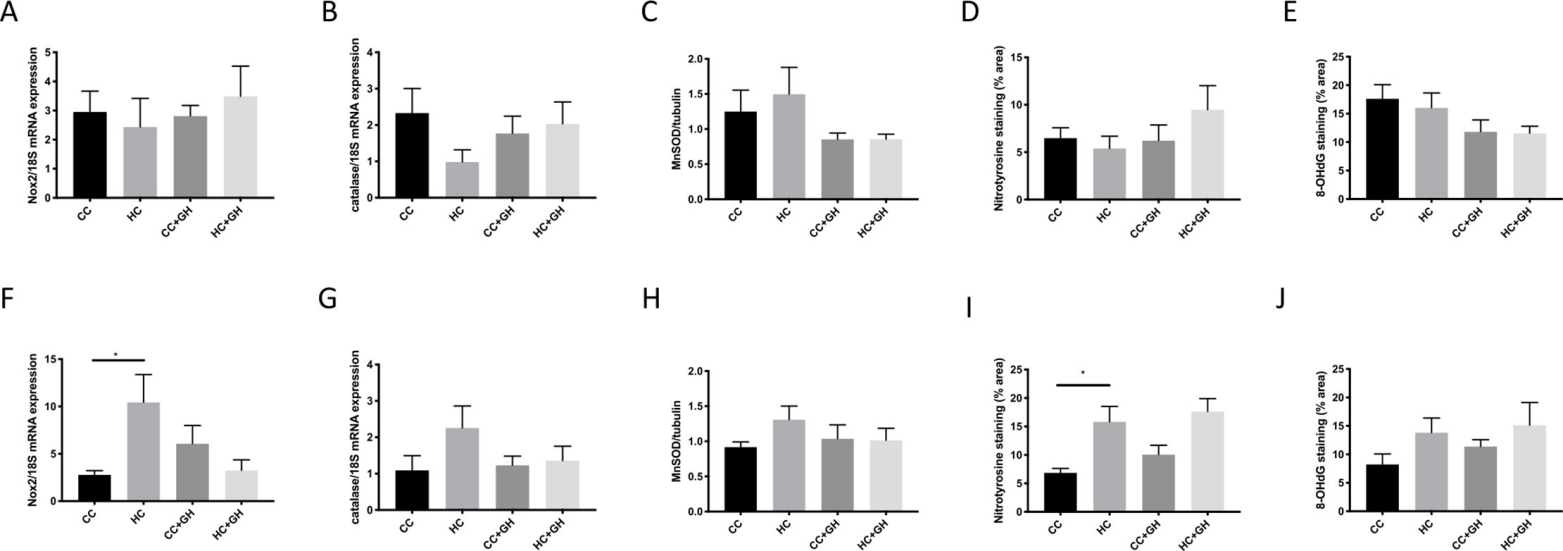
Markers of renal oxidative stress. (A) Nox2 mRNA expression at week 9, (B) catalase mRNA expression at week 9, (C) MnSOD expression measured by western blotting at week 9, (D) immunohistochemistry for nitrotyrosine at week 9, (E) immunohistochemistry for 8-OHdG at week 9, (F) Nox2 mRNA expression at week 32, (G) catalase mRNA expression at week 32, (H) MnSOD expression measured by western blotting at week 32, (I) immunohistochemistry for nitrotyrosine at week 32, (J) immunohistochemistry for 8-OHdG at week 32. Results expressed as mean ± SEM, n = 6. *p<0.05.

At week 9, maternal obesity did not affect oxidative stress markers in the kidneys of offspring. mRNA expression of NADPH oxidase (Nox)-2 and catalase were unaffected by exposure to maternal obesity or hydralazine (Fig 12A and 12B). There were no differences between groups in kidney expression of MnSOD (Fig 12C). Renal nitrotyrosine was not significantly differently expressed between offspring groups (Fig 12D). 8-OHdG was expressed at similar levels in offspring kidneys independent of maternal obesity and gestational hydralazine administration (Fig 12E).

At week 32, mRNA expression of Nox2 was significantly increased above control levels in HC (p<0.05, and hydralazine treatment tended to attenuate the mRNA expression of Nox2 in HC+GH compared to HC (p = 0.05, Fig 12F). Catalase mRNA expression was similar between HC and CC and remained unaffected by hydralazine exposure (Fig 12G). Expression of renal MnSOD tended to increase in HC compared to CC (p = 0.09), although gestational hydralazine was not protective (Fig 12H). There was significantly higher expression of renal nitrotyrosine in HC compared to CC (p<0.05), however gestational hydralazine exposure did not mitigate the effect of maternal obesity on renal nitrotyrosine (Fig 12I). 8-OHdG expression was similar between offspring groups, regardless of exposure to maternal obesity or hydralazine (Fig 12J).

### Effect of maternal obesity on offspring kidney inflammation

**Fig 13:**
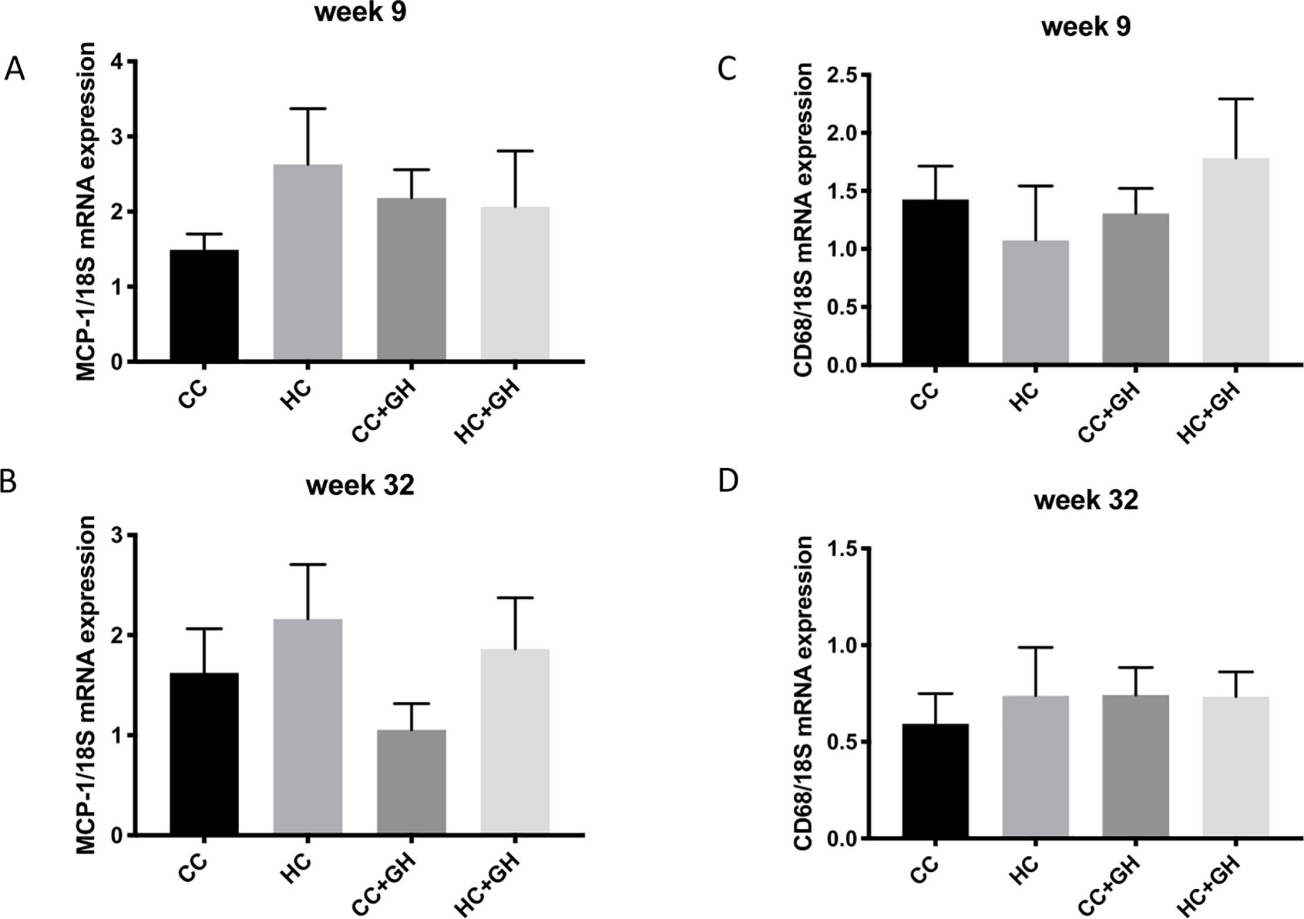
Kidney inflammation. (A, B) Renal MCP-1 mRNA expression in offspring at week 9 and week 32, (C, D) Renal CD68 mRNA expression in offspring at week 9 and week 32. Results expressed as mean ± SEM, n = 6.

Monocyte chemoattractant protein-1 (MCP-1) and CD68 were assessed in kidney tissue as markers of inflammation. At week 9, MCP-1 mRNA expression was similar between all offspring groups (Fig 13A). Similarly, at week 32, MCP-1 mRNA expression was unaffected by exposure to maternal obesity or hydralazine during gestation (Fig 13B). For both week 9 and week 32 offspring, CD68 mRNA expression was similar between all groups (Fig 13C and 13D). These results suggest that the deleterious renal effects of maternal obesity in offspring are independent of renal inflammation.

## Discussion

In this study, we observed that maternal obesity induces fibrotic markers of CKD in week 32 offspring. Gestational administration of low-dose hydralazine to mothers prevented the upregulation of several renal fibrotic markers at week 32 in offspring exposed to maternal obesity. Interestingly, protective effects of maternal hydralazine were observed at both the early time point of 9 weeks, and the later time point of 32 weeks. At postnatal week 9, offspring of hydralazine-treated obese mothers showed decreased renal expression of collagen III protein, compared to the offspring of saline-treated obese mothers. Further, week 9 offspring of hydralazine-treated obese mothers had attenuated expression of renal collagen IV protein, when compared with the offspring of untreated obese mothers. By week 32, offspring exposed to both maternal obesity and gestational hydralazine had lower protein expression of both collagen IV and fibronectin than offspring of saline-treated obese mothers. Further, collagen III mRNA expression was significantly reduced in the kidneys of week 32 offspring from hydralazine-treated obese mothers. Together, these results suggest that hydralazine administration during pregnancy has a powerful role in interrupting some of the adverse fetal programming effects of maternal obesity on renal fibrosis development in the offspring.

Consistent with previous studies conducted within our laboratory, maternal obesity was demonstrated to exert adverse renal effects on offspring, which persisted well into adulthood [5, 9, 10, 33]. In adult offspring at postnatal week 32, maternal obesity promoted renal pathology, specifically, albuminuria and glomerulosclerosis. Albuminuria is regarded as one of the earliest signs of asymptomatic kidney damage [34], reflecting glomerular damage and destruction of the integrity of the glomerular filtration barrier [35]. As albuminuria progresses, increased structural injury to podocytes occurs, resulting in glomerulosclerosis [36]. Although we observed only a relatively mild degree of glomerulosclerosis across all adult offspring groups, increased glomerulosclerosis was observed in offspring exposed to maternal obesity. Adult offspring of obese mothers also had elevated markers of renal fibrosis, with evidence of increased extracellular matrix components including collagen III, collagen IV and fibronectin.

Differences in biometric and metabolic parameters were identified between offspring of obese versus lean mothers at week 32. Although total body weight was similar between all offspring groups at this timepoint, offspring exposed to maternal obesity had higher kidney weight relative to total body weight, consistent with renal hypertrophy. Further, relative to total body weight, offspring of HFD-fed mothers had significantly higher liver weight. This is consistent with our previous study where liver weight was increased with increased lipid deposition [6]. This suggests a role of maternal obesity in disturbing offspring lipid metabolism. In the absence of postnatal HFD consumption, abdominal fat mass was similar between offspring from HFD-fed and chow-fed dams except for the epidydimal fat. This is expected as metabolic disorders due to maternal HFD consumption are more prominent with a second insult, such as postnatal HFD consumption. The reduction in epididymal fat may be related to impaired reproductive function due to maternal HFD consumption [37]. There were minimal differences in anthropometric, metabolic or renal endpoints at postnatal week 9, equivalent to late adolescence. This leads to the likely conclusion that for many of the parameters assessed, the week 9 time point was simply too early to appreciate significant renal or biometric changes.

Hydralazine is an arteriolar vasodilator which has an antihypertensive effect and has been in clinical use since the 1950s [25]. It is now mostly used in the management of resistant hypertension and hypertensive disorders of pregnancy [27]. Although it is considered safe overall in pregnancy, its use in the first trimester is currently not recommended [38]. Previous models of renal fibrosis have demonstrated a renoprotective effect of hydralazine via epigenetic mechanisms [23]. In our model, we observed at week 32 that gestational hydralazine was associated with significant reductions in renal global DNA methylation in offspring of both chow-fed and HFD-fed dams, also suggesting a role of epigenetic regulation. Surprisingly, there were no differences in renal global DNA methylation at week 9. This does not necessarily mean that there were no epigenetic changes at 9 weeks, as global DNA methylation represents the net effect of hyper- and hypomethylated genes. Additional experiments addressing whole genome methylation would be required to identify individual genes that are hyper- or hypomethylated and confirm whether hydralazine’s epigenetic effects are present from the early timepoint. We observed that maternal obesity also significantly increases mRNA expression of both DNMT1 and DNMT3a (enzymes which catalyse DNA methylation [39]) in the kidneys of adult offspring at week 32, and that hydralazine administration to obese mothers during gestation significantly ameliorated these effects. Hydralazine has previously been shown to inhibit methyltransferase activity in cancer cell cultures, in a dose dependent manner [40]. In experimental models of renal fibrosis involving unilateral ureteral obstruction and folic acid nephropathy, Tampe et al. demonstrated that the anti-fibrotic effects of low-dose hydralazine occurred through demethylation of the *RASAL1* promoter, mediated by induction of Tet3 hydroxymethylation [21, 23]. In the context of renal fibrosis, hydralazine was observed to downregulate mRNA expression of DNMT1 and induce expression of DNMT3a and Tet3 [21, 23]. While we observed a similar effect of maternal hydralazine on adult offspring renal DNMT1 mRNA expression, our results for DNMT3a and Tet3 were in the opposite direction. These varying observations are most likely explained by differences between the animal models used, and the fact that in our model, DNMTs and Tet3 were not assessed in animals who directly received hydralazine, but rather, in the subsequent generation. While these findings help support an epigenetic basis of hydralazine action, further studies are required to determine the differential methylation of genes in offspring exposed to maternal obesity with and without hydralazine. Although no prior studies have shown epigenetic regulation of extracellular matrix proteins by hydralazine, our results suggest that hydralazine may have a clinical role in mitigating the adverse offspring renal outcomes associated with maternal obesity.

We observed some evidence of increased renal oxidative stress in offspring of obese mothers at week 32, aligning with our previous work demonstrating oxidative stress as a key mechanism by which maternal obesity programs offspring CKD [5, 9, 33]. Nitrotyrosine, a stable marker of oxidative stress in inflammatory diseases [41], was observed to be expressed at higher levels in the kidneys of offspring of obese mothers, than in those from lean mothers. Exposure to maternal obesity during gestation also increased offspring renal mRNA expression of Nox2, an isoform of NADPH oxidase which produces superoxide [42], and has previously been demonstrated to correlate with superoxide levels [43]. Hydralazine administration to obese mothers during pregnancy tended to decrease renal mRNA expression of Nox2 in week 32 offspring. It has previously been suggested that hydralazine may exert antioxidant effects, either by preventing superoxide formation through Nox inhibition, or by directly scavenging reactive oxygen species [44]. Although our data demonstrated a trend of decreased Nox2 expression with gestational hydralazine exposure, additional experiments to assess the superoxide level are required to confirm whether hydralazine’s effects were through superoxide changes in our maternal obesity model.

In summary, we demonstrated that maternal obesity has deleterious renal consequences for the offspring in rodents, exerting profound effects on the fetal programming of CKD. In the offspring of obese mothers, we observed increased albuminuria, glomerulosclerosis, renal fibrosis and renal markers of oxidative stress, which worsened with advancing age of the offspring. Administration of low-dose hydralazine to obese mothers during pregnancy was associated with reductions in renal fibrotic markers in offspring. This is the first study using an intergenerational model to demonstrate that hydralazine may have utility in preventing maternal obesity from programming CKD in the subsequent generation. The mechanism underlying hydralazine’s action remains unclear, however our data support that it may be mediated via DNA demethylation effects. The repurposing of hydralazine as a novel therapy to reduce maternal obesity-induced CKD warrants further investigation, especially given its overall safety profile in the setting of pregnancy.

## Supporting information

S1 Checklist The ARRIVE guidelines 2.0: Author checklist. (PDF)

S1 Raw images (PDF)
