# Supplementary material for: Low-dose hydralazine during gestation reduces renal fibrosis in rodent offspring exposed to maternal high fat diet
Source: PLoS One. 2021 Mar 18;16(3):e0248854. doi: 10.1371/journal.pone.0248854 (PMC7971884; doi:10.1371/journal.pone.0248854)
Supplement: S1 Raw images — (PDF) [file pone.0248854.s002.pdf]

Week 9 Collagen III

Used in Fig 6B

Captured by ImageQuant LAS 4000 (Fujifilm, Tokyo, Japan)

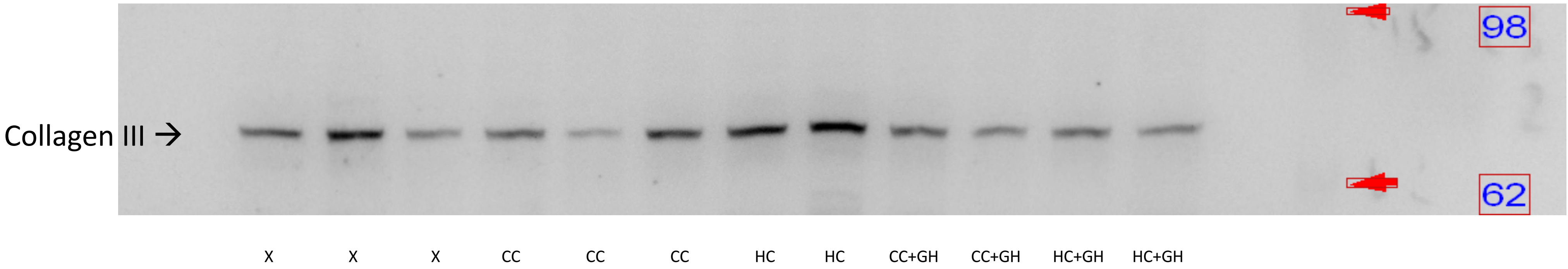

X= lanes not included in final figures
(treatment groups irrelevant to this manuscript)

α-tubulin for week 9 Collagen III

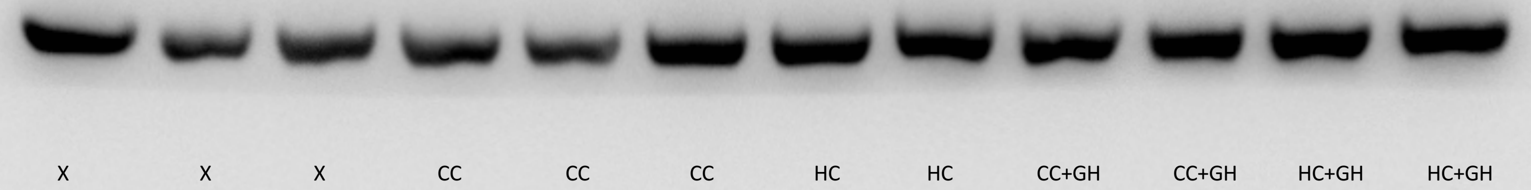

Used in Fig 6B

Captured by ImageQuant LAS 4000 (Fujifilm, Tokyo, Japan)

Week 32 Collagen III

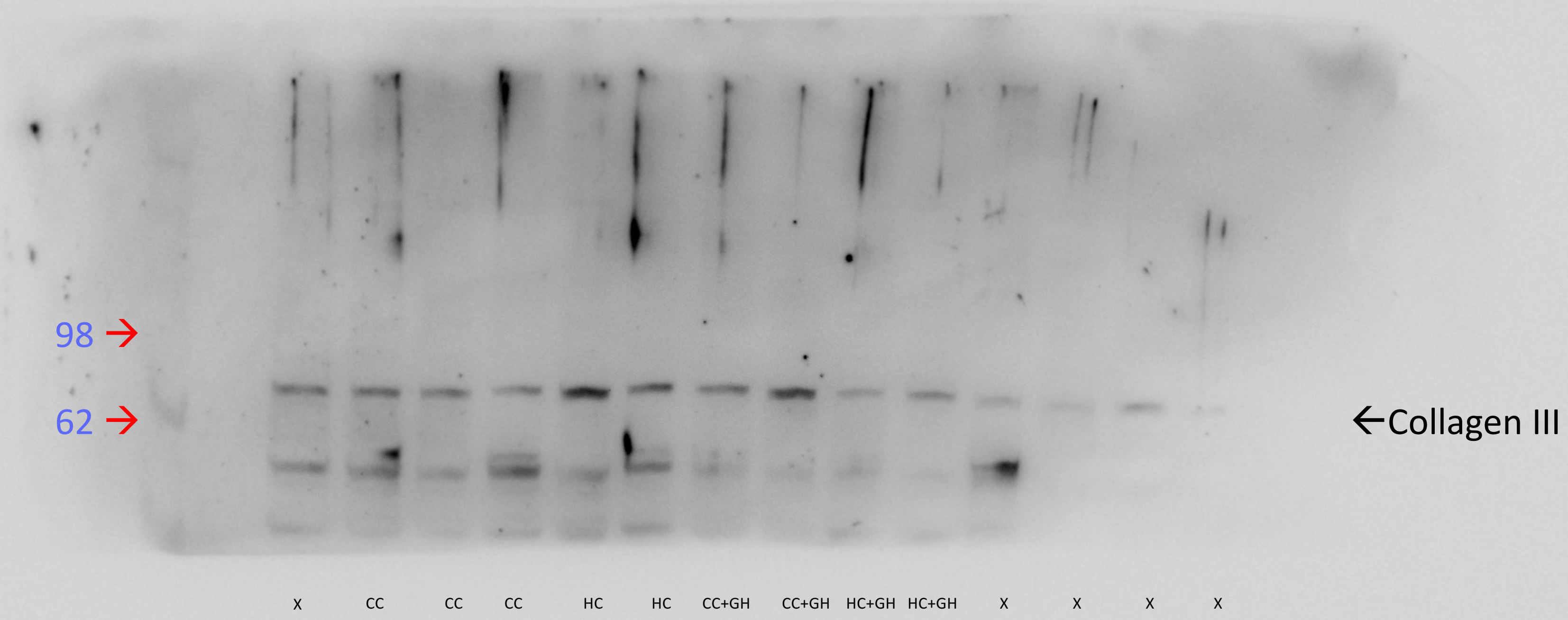

Used in Fig 6D

Captured by ImageQuant LAS 4000 (Fujifilm, Tokyo, Japan)

α-tubulin for week 32 Collagen III

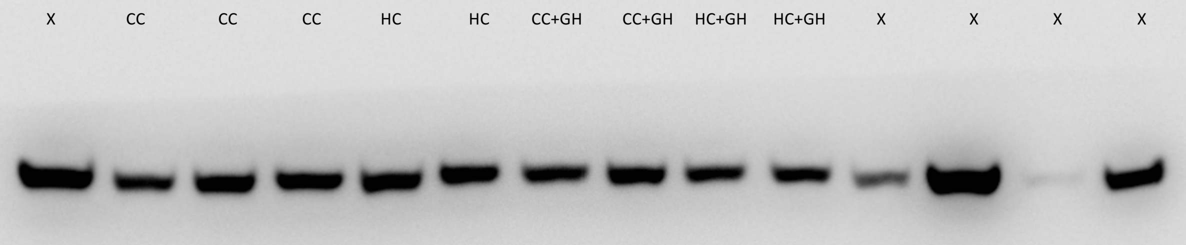

Captured by ImageQuant LAS 4000 (Fujifilm, Tokyo, Japan)

Used in Fig 6D

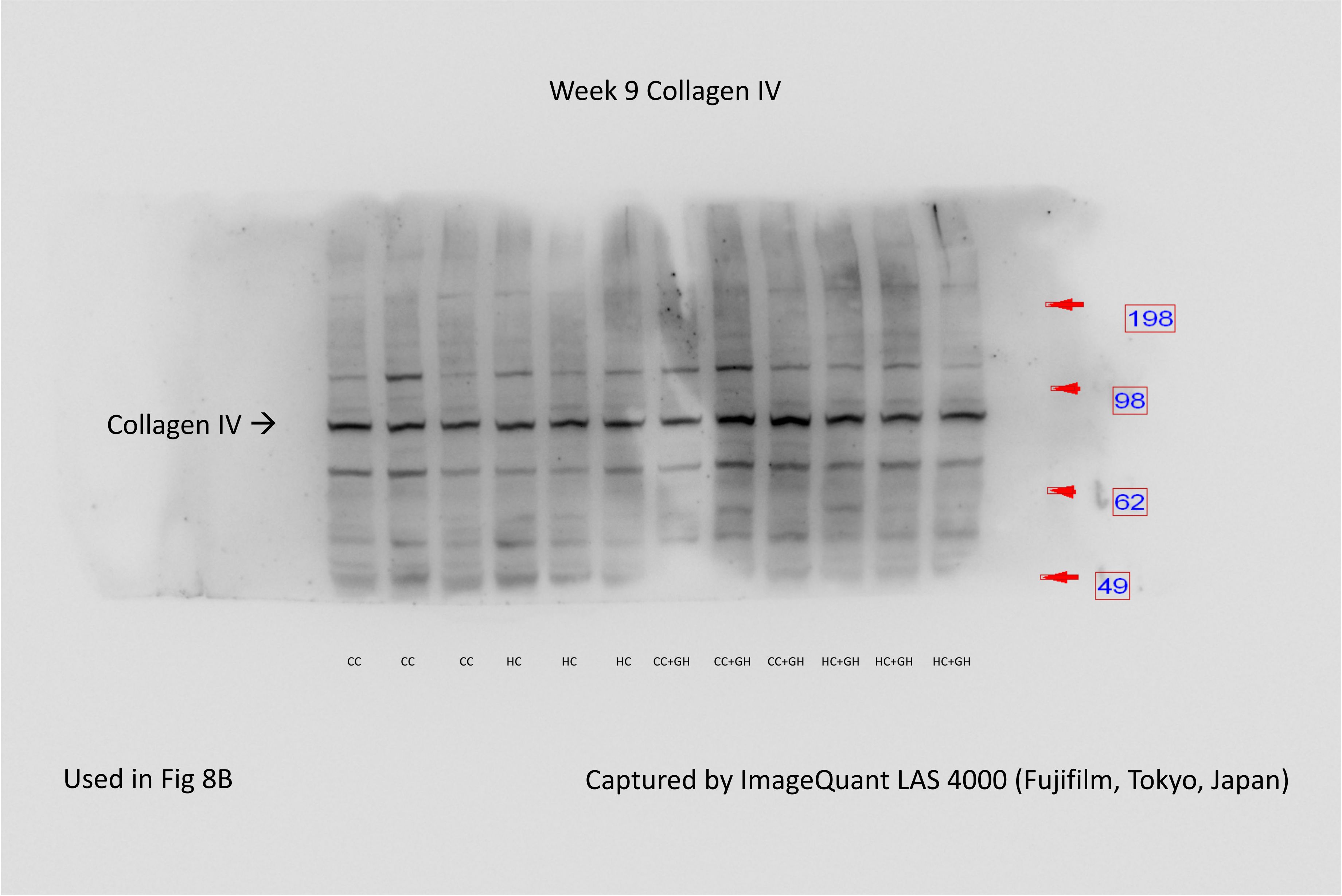

Week 9 Collagen IV
Collagen IV →
198
98
62
49
CC
CC
CC
HC
HC
HC
CC+GH
CC+GH
CC+GH
HC+GH
HC+GH
HC+GH
Used in Fig 8B
Captured by ImageQuant LAS 4000 (Fujifilm, Tokyo, Japan)

α-tubulin for week 9 Collagen IV

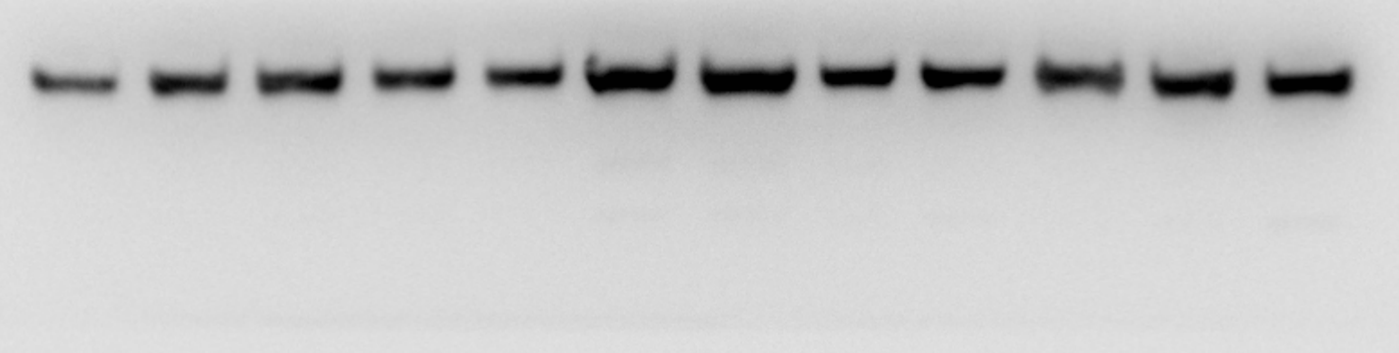

Used in Fig 8B

Captured by ImageQuant LAS 4000 (Fujifilm, Tokyo, Japan)

Week 32 Collagen IV

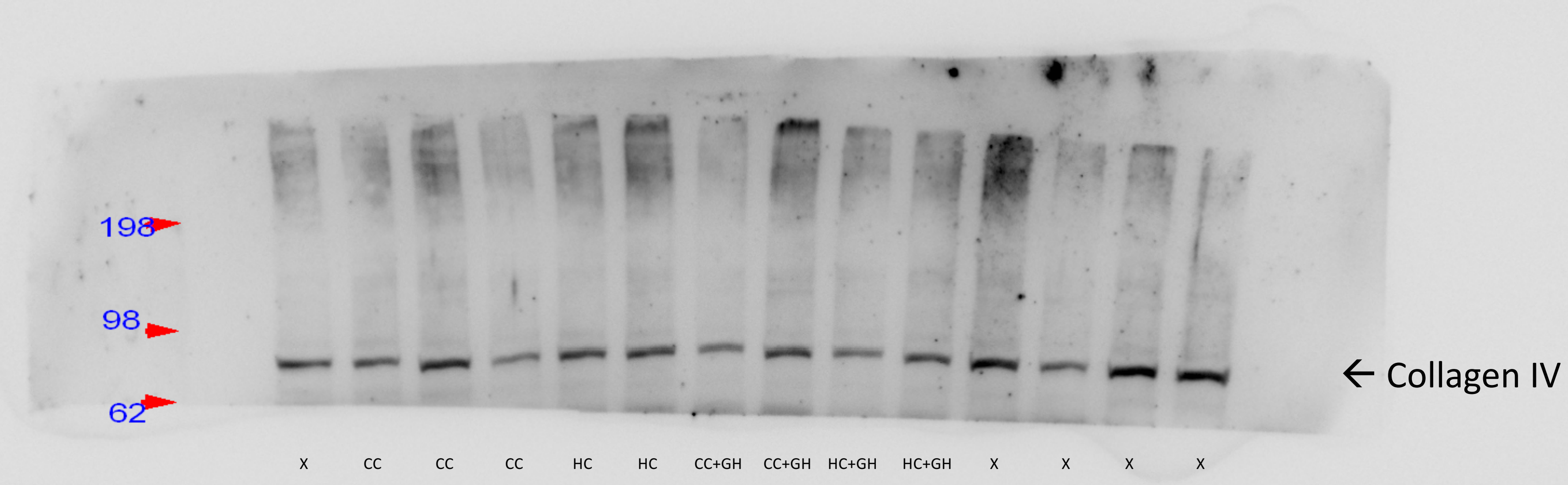

Used in Fig 8D

Captured by ImageQuant LAS 4000 (Fujifilm, Tokyo, Japan)

α-tubulin for week 32 Collagen IV

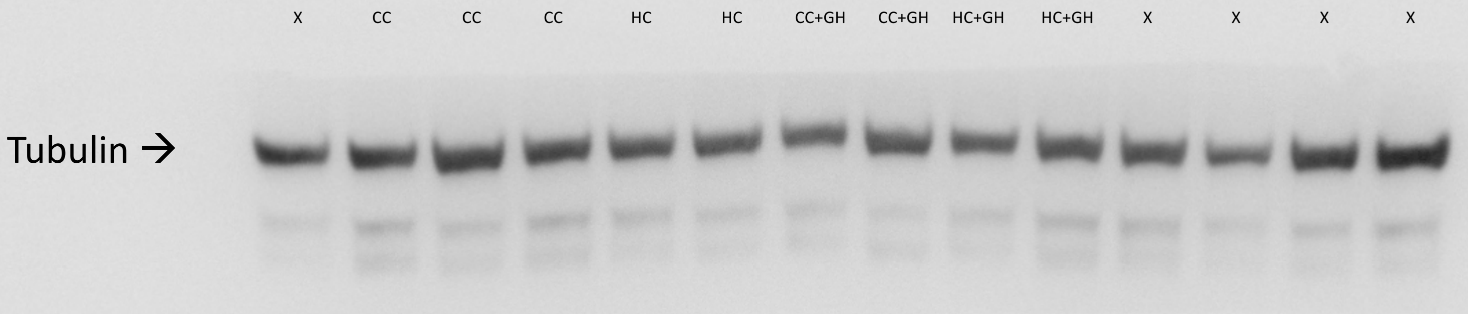

Used in Fig 8D

Captured by ImageQuant LAS 4000 (Fujifilm, Tokyo, Japan)

Week 9 Fibronectin

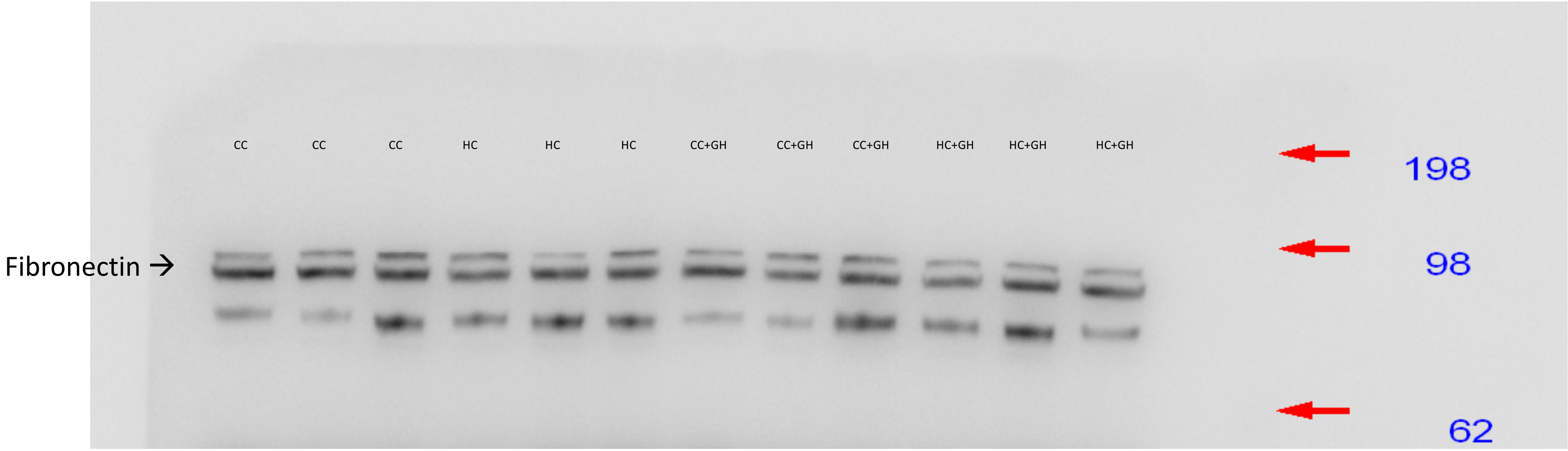

Used in Fig 10B

Captured by ImageQuant LAS 4000 (Fujifilm, Tokyo, Japan)

α-tubulin for week 9 Fibronectin

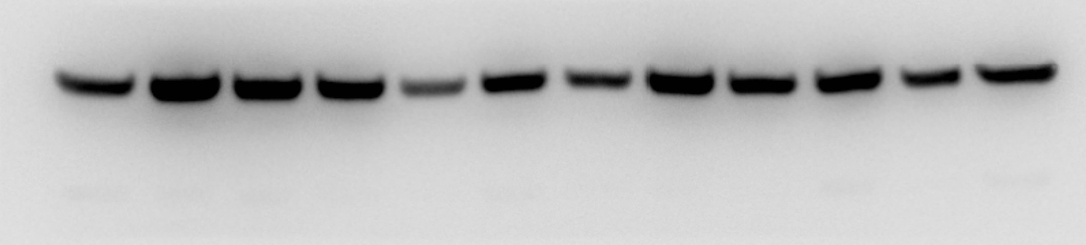

Used in Fig 10B

Captured by ImageQuant LAS 4000 (Fujifilm, Tokyo, Japan)

Week 32 Fibronectin

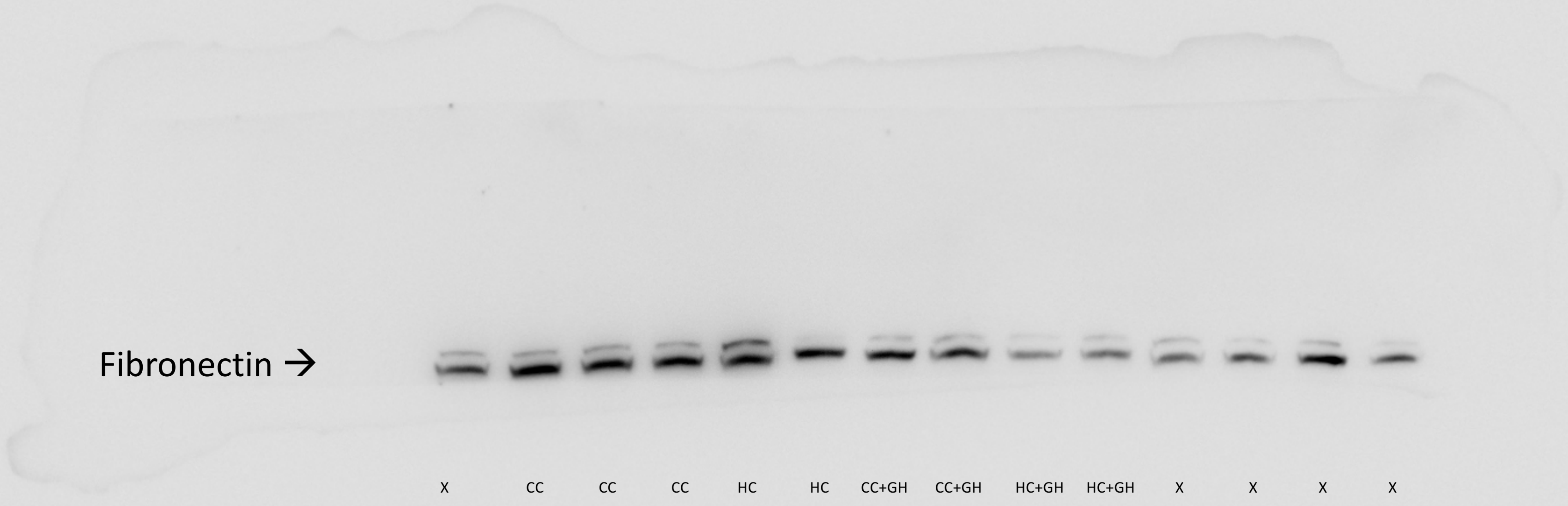

Used in Fig 10D

Captured by ImageQuant LAS 4000 (Fujifilm, Tokyo, Japan)

α-tubulin for week 32 Fibronectin

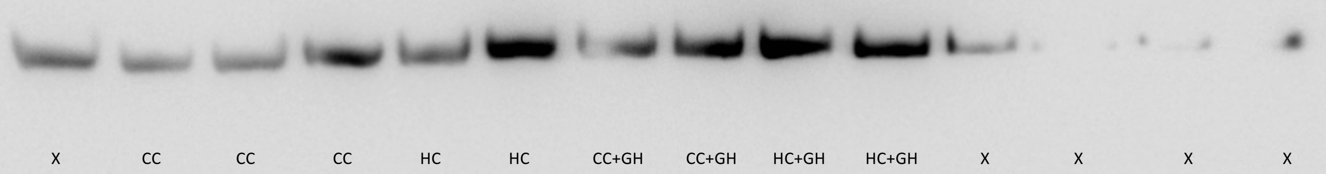

Used in Fig 10D

Captured by ImageQuant LAS 4000 (Fujifilm, Tokyo, Japan)
